# Comparison of Long-term Performance of Bioprosthetic Aortic Valves in Sweden From 2003 to 2018

**DOI:** 10.1001/jamanetworkopen.2022.0962

**Published:** 2022-03-07

**Authors:** Michael Persson, Natalie Glaser, Johan Nilsson, Örjan Friberg, Anders Franco-Cereceda, Ulrik Sartipy

**Affiliations:** 1 Department of Molecular Medicine and Surgery, Karolinska Institutet, Stockholm, Sweden; 2 Department of Cardiothoracic Surgery, Karolinska University Hospital, Stockholm, Sweden; 3 Department of Cardiology, Stockholm South General Hospital, Stockholm, Sweden; 4 Department of Translational Medicine, Cardiothoracic Surgery and Bioinformatics, Lund University, Lund, Sweden; 5 Department of Cardiothoracic and Vascular Surgery, Skåne University Hospital, Lund, Sweden; 6 Department of Cardiothoracic and Vascular Surgery, Örebro University Hospital, Örebro, Sweden

## Abstract

**Importance:**

The performance of bioprosthetic aortic valves is usually assessed in single valve models or head-to-head comparisons. National databases or registries offer the opportunity to investigate all available valve models in the population and allows for a comparative assessment of their performance.

**Objective:**

To analyze the long-term rates of reintervention, all-cause mortality, and heart failure hospitalization associated with commonly used bioprosthetic aortic valves and to identify valve model groups with deviation in clinical performance.

**Design, Setting, and Participants:**

This population-based, nationwide cohort study included all adult patients who underwent surgical aortic valve replacement (with or without concomitant coronary artery bypass surgery or ascending aortic surgery) in Sweden between January 1, 2003, and December 31, 2018. Patients were identified from the SWEDEHEART (Swedish Web-System for Enhancement and Development of Evidence-Based Care in Heart Disease Evaluated According to Recommended Therapies) registry. Patients with concomitant valve surgery, previous cardiac surgery, and previous transcatheter valve replacement were excluded. Follow-up was complete for all participants. Data were analyzed from March 9, 2020, to October 12, 2021.

**Exposures:**

Primary surgical aortic valve replacement with the Perimount, Mosaic/Hancock, Biocor/Epic, Mitroflow/Crown, Soprano, and Trifecta valve models.

**Main Outcomes and Measures:**

The primary outcome was cumulative incidence of reintervention, defined as a subsequent aortic valve operation or transcatheter valve replacement. Secondary outcomes were all-cause mortality and heart failure hospitalization. Regression standardization and flexible parametric survival models were used to account for intergroup differences. Mean follow-up time was 7.1 years, and maximum follow-up time was 16.0 years.

**Results:**

A total of 16 983 patients (mean [SD] age, 72.6 [8.5] years; 10 685 men [62.9%]) were included in the analysis. The Perimount valve model group had the lowest and the Mitroflow/Crown valve model group had the highest cumulative incidence of reintervention. The estimated cumulative incidence of reintervention at 10 years was 3.6% (95% CI, 3.1%-4.2%) in the Perimount valve model group and 12.2% (95% CI, 9.8%-15.1%) in the Mitroflow/Crown valve model group. The estimated incidence of reintervention at 10 years was 9.3% (95% CI, 7.3%-11.3%) in the Soprano valve model group.

**Conclusions and Relevance:**

Results of this study showed that the Perimount valve was the most commonly used and had the lowest incidence of reintervention, all-cause mortality, and heart failure hospitalization, whereas the Mitroflow/Crown valve had the highest rates. These findings highlight the need for clinical vigilance in patients who receive either a Soprano or Mitroflow/Crown aortic bioprosthesis.

## Key Points

**Question:**

Are the rates of reintervention, all-cause mortality, and heart failure hospitalization different between commonly used bioprosthetic aortic valves?

**Findings:**

In this cohort study of 16 983 patients who underwent primary surgical aortic valve replacement in Sweden between 2003 and 2018, the Perimount valve model group had a significantly lower cumulative incidence of reintervention, all-cause mortality, and heart failure hospitalization, whereas the Mitroflow/Crown valve model group had significantly higher rates; the Soprano valve model group also had an increased incidence of reintervention.

**Meaning:**

Findings from this study support the widespread use of the Perimount valve and highlight the need for clinical vigilance in patients who have received either a Soprano or a Mitroflow/Crown valve.

## Teaser

This cohort study assesses the reintervention, all-cause mortality, and heart failure hospitalization rates for each type of bioprosthetic aortic valve used in Sweden.

## Introduction

Bioprosthetic aortic valves are currently the most common type of surgical valve.^1^ Since bioprosthetic aortic valve replacement was first described in the 1960s, a wide range of different bioprosthetic models have been developed to improve patient outcomes.^2,3,4^ Different xenograft materials have been used, such as porcine or bovine pericardium or valve tissue.^5,6^ Efforts to optimize the hemodynamic performance of bioprosthetic valves have included the supra-annular approach, external wrapping of leaflets around stents, and stentless designs.^7,8,9,10^ In attempts to improve the longevity of valves, different methods of tissue preservation and anticalcification treatments were developed.^11^

Previous studies on bioprostheses were usually performed with single valve models or were head-to-head comparisons of 2 models.^12,13^ The Valve Academic Research Consortium 3, which recently released end point definitions for aortic valve research, recommends clinically and economically meaningful end points, including all-cause mortality, reintervention, and hospitalization.^14^ Hickey et al^15^ proposed using postmarket surveillance to compare a larger set of valve models to identify unexpected patterns in performance. The benefit of this approach is the direct comparison of multiple models from the same population that can help identify deviance in performance. The availability of national health data registries in Sweden enables such large, nationwide comparative studies.

To our knowledge, no study has assessed the performance of bioprosthetic valves that are used in Sweden with a similar multiple comparison approach used by Hickey et al.^15^ In the current study, we aimed to assess the long-term rates of reintervention, all-cause mortality, and heart failure hospitalization associated with commonly used bioprosthetic aortic valves and to identify model groups with deviation in clinical performance that may warrant further attention.

## Methods

This observational, population-based, nationwide cohort study was conducted in Sweden. Ethical permission was approved by the Swedish Ethical Review Authority, which waived the requirement for informed consent because the data were deidentified. Study reporting followed the Strengthening the Reporting of Observational Studies in Epidemiology (STROBE) reporting guideline and the Reporting of Studies Conducted Using Observational Routinely Collected Health Data (RECORD) guideline.^16,17^

### Study Population and Data Sources 

All adult patients in Sweden who underwent surgical bioprosthetic aortic valve replacement between January 1, 2003, and December 31, 2018, with or without concomitant coronary artery bypass grafting or ascending aortic surgery were included in the study. Patients were excluded if they met 1 of the following criteria: younger than 18 years, previous cardiac surgery, previous transcatheter aortic valve replacement (TAVR), concomitant surgery on another valve, use of deep hypothermia and circulatory arrest, undetermined prosthesis type, or use of a stentless or rapid deployment valve. During the study period, cardiac surgery was performed in 8 centers in Sweden (Karolinska University Hospital, Stockholm; Uppsala University Hospital, Uppsala; University Hospital of Umeå, Umeå; Skåne University Hospital, Lund; Linköping University Hospital, Linköping; Örebro University Hospital, Örebo; Hospital of Blekinge, Karlskrona; and Sahlgrenska University Hospital, Gothenburg). Reported data on cardiac surgery volumes and quality of care are available through the SWEDEHEART (Swedish Web-System for Enhancement and Development of Evidence-Based Care in Heart Disease Evaluated According to Recommended Therapies) registry, and clinical outcomes are similar across all 8 centers.

The SWEDEHEART registry was used to identify the study population.^18^ This nationwide registry consists of several subregistries, including the Swedish Cardiac Surgery Registry, which collects preoperative, perioperative, and postoperative data on all patients who undergo cardiac surgery in Sweden. A 2018 review showed that the Swedish Cardiac Surgery Registry had excellent coverage and high reliability.^19^ The Swedish National Inpatient Register was used to identify baseline characteristics of preexisting morbidity and the characteristics of heart failure and TAVR reintervention at follow-up.^20^ The diagnosis of heart failure in the National Inpatient Register has been validated and has high reliability.^21^ Baseline socioeconomic characteristics were obtained from the Longitudinal Integrated Database for Health Insurance and Labour Market Studies Register, which is maintained by Statistics Sweden.^22^ Cross-linking of individual-level data was possible because of the Swedish personal identity number.^23^ These national registers have been described elsewhere.^1^

### Exposures, Outcomes, and Missing Data

In Sweden, valve model selection is largely hospital based owing to a local public procurement process at each surgical center. Valve model groups were classified as follows: Perimount (Perimount 2900, Perimount Magna, and Perimount Magna Ease), Mosaic/Hancock (Hancock II, Hancock II Ultra, and Mosaic and Mosaic Ultra), Biocor/Epic (Epic, Epic Supra, and Biocor), Mitroflow/Crown (Mitroflow and Crown PRT), Soprano (Soprano Armonia Valve), and Trifecta (Trifecta). A few valves (eg, Inspiris Resilia) were excluded from further analysis because of the small number of valves. The frequencies of different valve models within each group are shown in eTable 1 in the Supplement.

The primary outcome was the cumulative incidence of reintervention. Reintervention was defined as a subsequent aortic valve operation or a valve-in-valve TAVR procedure. Secondary outcomes were all-cause mortality and heart failure hospitalization.

There were missing data for the following variables: body mass index (7.4%), estimated glomerular filtration rate (1.7%), educational level (1.2%), left ventricular ejection fraction (0.9%), emergent operation (0.8%), valve size (0.3%), and disposable family income (0.02%). Missing data were handled with the Classification and Regression Tree estimation and imputation approach.^24^ Swedish regulations prohibit the recording of race and ethnicity data; these data were not collected.

### Statistical Analysis

Baseline characteristics were described as frequencies (percentages) for categorical variables and as means (SDs) for continuous variables. The time to event was calculated as the time in days from the date of operation until the date of reintervention, death, heart failure hospitalization, or end of follow-up (December 31, 2018), whichever occurred first. Significance was decided as nonoverlapping 95% CIs between the estimates. The Aalen-Johansen estimator was used to obtain the crude cumulative incidence while accounting for the competing risk of death.^25^ Cumulative incidence of all-cause mortality was estimated with the Kaplan-Meier method. Age- and sex-adjusted incidence rates were obtained from a Poisson model.

The standardized cumulative incidence of reintervention and heart failure hospitalization was calculated by flexible hazard-based regression standardization as described by Kipourou et al.^26^ The results from this method can be interpreted as the estimated cumulative incidence curve if the entire population receives the same respective valve. For example, if the entire population were implanted with a Perimount valve, 3.6% of these individuals may have a reintervention in 10 years. This calculation adjusts for the population distribution of covariates while accounting for the competing risk of death. Standardized cumulative survival was estimated using flexible parametric regression standardization.^27,28^ The resulting survival curve is an estimate of population survival if the entire population receives the same respective valve. This estimation adjusts for the population distribution of covariates. Model selection was performed using a combination of clinical judgment and a backward selection strategy. A detailed description of regression standardization and the final models are provided in the eMethods in the Supplement.

We repeated the main analyses in a subset of patients who underwent isolated aortic valve replacement as well as on model level rather than model group. Data management and statistical analyses were performed using R, version 4.0.4 with the survival, ggplot, mexhaz, stdReg, and rstpm2 packages (R Foundation for Statistical Computing).^29,30,31,32,33^ Data were analyzed from March 9, 2020, to October 12, 2021.

## Results

We identified 16 983 patients who underwent bioprosthetic aortic valve replacement from 2003 to 2018. Patients had a mean (SD) age of 72.6 (8.5) years and included 10 685 men (62.9%) and 6298 women (37.1%). Overall, there were small but potentially important differences among patients in the valve model groups. The mean (SD) patient age was 71.4 (8.9) years in the Perimount valve model group compared with 76.1 (6.6) years in the Biocor/Epic valve model group. Another important difference was the use of small valve sizes. In the Mosaic/Hancock valve model group, 27.6% of the patients (n = 340) received a 19- or 21-mm valve compared with 62.5% of the patients (n = 120) in the Trifecta valve model group. The most common valve model in the study population was Perimount (11 269 [66%]), and its use increased during the study period. The least common valve model was Trifecta (192 [1%]), in part because no Trifecta valves were implanted before 2010. Baseline characteristics and intergroup differences are shown in Table 1. Baseline characteristics stratfied by outcome are presented in eTable 4 in the Supplement. Use by valve model group and year is shown in eFigure 1 in the Supplement. The distributions of age, valve size, and left ventricular ejection fraction in each valve model group are shown in eFigures 2 to 4 in the Supplement.

**Table 1.** Baseline Characteristics of Patients Who Underwent Bioprosthetic Aortic Valve Replacement in Sweden From 2003 to 2018

Variable	Valve model group						
	Overall	Perimount	Mosaic/Hancock	Biocor/Epic	Mitroflow/Crown	Soprano	Trifecta
Total No. of patients	16 983	11 269	1235	1670	1643	974	192
Age, mean (SD), y	72.6 (8.5)	71.4 (8.9)	75.0 (7.3)	76.1 (6.6)	74.8 (6.7)	73.5 (8.4)	73.6 (6.3)
Female sex	6298 (37.1)	4043 (35.9)	469 (38.0)	698 (41.8)	615 (37.4)	359 (36.9)	114 (59.4)
Male sex	10 685 (62.9)	7226 (64.1)	766 (62.0)	972 (68.2)	1028 (62.6)	615 (63.1)	78 (40.6)
Married	10 890 (64.1)	7197 (63.9)	770 (62.3)	1086 (65.0)	1075 (65.4)	647 (66.4)	115 (59.9)
BMI							
<18.5	147 (0.9)	102 (0.9)	9 (0.8)	12 (0.8)	14 (1.1)	6 (0.7)	4 (2.1)
18.5-24.9	5334 (33.9)	3600 (33.2)	436 (36.4)	504 (35.7)	411 (33.1)	318 (38.0)	65 (34.6)
25-29.9	6690 (42.5)	4599 (42.4)	521 (43.5)	606 (42.9)	525 (42.2)	359 (42.9)	80 (42.6)
≥30	3562 (22.6)	2556 (23.5)	231 (19.3)	290 (20.5)	293 (23.6)	153 (18.3)	39 (20.7)
Educational level, y							
<10	7222 (43.0)	4547 (40.8)	574 (47.0)	890 (54.2)	700 (43.1)	448 (46.6)	63 (33.2)
10-12	6320 (37.7)	4313 (38.7)	443 (36.3)	535 (32.6)	604 (37.2)	344 (35.8)	81 (42.6)
>12	3241 (19.3)	2286 (20.5)	203 (16.6)	217 (13.2)	319 (19.7)	170 (17.7)	46 (24.2)
Household income							
Quartile 1 (lowest)	4245 (25.0)	2553 (22.7)	366 (29.6)	596 (35.7)	413 (25.2)	283 (29.1)	34 (17.7)
Quartile 2	4245 (25.0)	2672 (23.7)	340 (27.5)	479 (28.7)	404 (24.6)	292 (30.0)	58 (30.2)
Quartile 3	4245 (25.0)	2914 (25.9)	283 (22.9)	357 (21.4)	429 (26.1)	209 (21.5)	53 (27.6)
Quartile 4 (highest)	4245 (25.0)	3129 (27.8)	246 (19.9)	237 (14.2)	396 (24.1)	190 (19.5)	47 (24.5)
Non-Nordic birth region	1016 (6.0)	663 (5.9)	64 (5.2)	99 (5.9)	108 (6.6)	76 (7.8)	6 (3.1)
LVEF, %							
<30	888 (5.3)	556 (5.0)	58 (4.7)	99 (6.0)	94 (5.8)	78 (8.1)	3 (1.6)
30-50	3725 (22.1)	2396 (21.4)	336 (27.2)	379 (23.1)	367 (22.5)	199 (20.6)	48 (25.0)
>50	12225 (72.6)	8222 (73.6)	840 (68.1)	1162 (70.9)	1170 (71.7)	690 (71.4)	141 (73.4)
Previous heart condition							
Myocardial infarction	2858 (16.8)	1688 (15.0)	226 (18.3)	355 (21.3)	355 (21.6)	197 (20.2)	37 (19.3)
Heart failure	3701 (21.8)	2343 (20.8)	250 (20.2)	440 (26.3)	400 (24.3)	236 (24.2)	32 (16.7)
Atrial fibrillation	3202 (18.9)	2076 (18.4)	207 (16.8)	350 (21.0)	341 (20.8)	196 (20.1)	32 (16.7)
Pacemaker/ICD	467 (2.7)	329 (2.9)	33 (2.7)	31 (1.9)	44 (2.7)	23 (2.4)	7 (3.6)
Previous PCI	1561 (9.2)	1058 (9.4)	102 (8.3)	122 (7.3)	163 (9.9)	91 (9.3)	25 (13.0)
Hyperlipidemia	3931 (23.1)	2699 (24.0)	290 (23.5)	302 (18.1)	363 (22.1)	207 (21.3)	70 (36.5)
Hypertension	9786 (57.6)	6571 (58.3)	666 (53.9)	849 (50.8)	1040 (63.3)	525 (53.9)	135 (70.3)
Previous endocarditis	816 (4.8)	606 (5.4)	49 (4.0)	44 (2.6)	70 (4.3)	38 (3.9)	9 (4.7)
Peripheral vascular disease	2243 (13.2)	1429 (12.7)	180 (14.6)	229 (13.7)	252 (15.3)	128 (13.1)	25 (13.0)
eGFR, mL/min/1.73 m^2^							
<30	480 (2.9)	277 (2.5)	28 (2.3)	69 (4.2)	82 (5.2)	18 (1.9)	6 (3.1)
30-44	1309 (7.8)	816 (7.4)	104 (8.5)	157 (9.5)	144 (9.1)	69 (7.2)	19 (9.9)
45-59	3120 (18.7)	2022 (18.3)	215 (17.6)	337 (20.4)	328 (20.6)	182 (18.9)	36 (18.8)
≥60	11 793 (70.6)	7963 (71.9)	878 (71.7)	1089 (65.9)	1036 (65.2)	696 (72.1)	131 (68.2)
COPD	1938 (11.4)	1166 (10.3)	150 (12.1)	208 (12.5)	262 (15.9)	133 (13.7)	19 (9.9)
Diabetes	3791 (22.3)	2482 (22.0)	249 (20.2)	368 (22.0)	426 (25.9)	209 (21.5)	57 (29.7)
Previous stroke	2105 (12.4)	1326 (11.8)	150 (12.1)	238 (14.3)	256 (15.6)	111 (11.4)	24 (12.5)
History of cancer	2911 (17.1)	1831 (16.2)	176 (14.3)	316 (18.9)	358 (21.8)	196 (20.1)	34 (17.7)
Hepatic disease	279 (1.6)	185 (1.6)	20 (1.6)	23 (1.4)	29 (1.8)	19 (2.0)	3 (1.6)
Alcohol dependence	500 (2.9)	334 (3.0)	35 (2.8)	34 (2.0)	55 (3.3)	36 (3.7)	6 (3.1)
Period of surgery, y							
2003-2008	4995 (29.4)	2842 (25.2)	537 (43.5)	880 (52.7)	265 (16.1)	471 (48.4)	0
2009-2013	6085 (35.8)	3690 (32.7)	594 (48.1)	736 (44.1)	501 (30.5)	499 (51.2)	65 (33.9)
2014-2018	5903 (34.8)	4737 (42.0)	104 (8.4)	54 (3.2)	877 (53.4)	4 (0.4)	127 (66.1)
Valve size, mm							
19-21	5772 (34.1)	3667 (32.7)	340 (27.6)	516 (31.0)	598 (36.4)	531 (54.7)	120 (62.5)
23	6515 (38.5)	4287 (38.2)	555 (45.1)	645 (38.8)	678 (41.3)	301 (31.0)	49 (25.5)
≥25	4639 (27.4)	3274 (29.2)	335 (27.2)	503 (30.2)	366 (22.3)	138 (14.2)	23 (12.0)
Concomitant CABG	6453 (38.0)	3893 (34.5)	571 (46.2)	789 (47.2)	739 (45.0)	401 (41.2)	60 (31.2)
Ascending aortic surgery	1441 (8.5)	1049 (9.3)	75 (6.1)	101 (6.0)	159 (9.7)	46 (4.7)	11 (5.7)
Emergent operation	289 (1.7)	184 (1.6)	23 (1.9)	23 (1.4)	41 (2.5)	15 (1.6)	3 (1.6)

Abbreviations: BMI, body mass index (calculated as weight in kilograms divided by height in meters squared); CABG, coronary artery bypass graft; COPD, chronic obstructive pulmonary disease; eGFR, estimated glomerular filtration rate; ICD, implantable cardioverter-defibrillator; LVEF, left ventricular ejection fraction; PCI, percutaneous coronary intervention.

### Reintervention

During a follow-up time of 120 768 person-years (mean follow-up: 7.1 years; maximum follow-up: 16.0 years), 520 patients underwent reintervention with surgical aortic valve replacement or TAVR. Crude and age- and sex-adjusted incidence rates are presented in the eResults and eTable 2 in the Supplement.

The crude cumulative incidence rates at 5, 10, and 15 years are shown in Table 2. The Perimount valve model group had the lowest crude cumulative incidence of reintervention at 10 years (3.0%; 95% CI, 2.6%-3.5%), but its cumulative incidence rate at 15 years was 5.1% (95% CI, 4.2%-6.0%). The Soprano valve model group had the highest crude cumulative incidence of reintervention at 10 years (9.3%; 95% CI, 7.3%-11.3%). Similar to the Soprano valve model group, the Mitroflow/Crown valve model group had a crude cumulative incidence at 10 years of 9.2% (95% CI, 6.9%-11.4%).

**Table 2.** Crude Cumulative Incidence of Reintervention, All-Cause Mortality, and Heart Failure Hospitalization at 5, 10, and 15 Years After Bioprosthetic Aortic Valve Replacement in Sweden From 2003 to 2018

Valve model group	Cumulative incidence, % (95% CI)		
	5 y	10 y	15 y
**Reintervention** ^a^			
Perimount	1.5 (1.3-1.8)	3.0 (2.6-3.5)	5.1 (4.2-6.0)
Mosaic/Hancock	1.5 (0.8-2.1)	3.5 (2.3-4.7)	4.9 (3.2-6.6)
Biocor/Epic	2.0 (1.3-2.7)	3.7 (2.8-4.7)	4.7 (3.4-6.0)
Mitroflow/Crown	3.5 (2.4-4.5)	9.2 (6.9-11.4)	9.8 (7.4-12.2)
Soprano	3.6 (2.4-4.8)	9.3 (7.3-11.3)	NA
Trifecta	2.2 (0.0-4.8)	NA	NA
**All-cause mortality** ^b^			
Perimount	18 (18-19)	44 (43-45)	72 (70-75)
Mosaic/Hancock	22 (20-24)	54 (50-57)	82 (78-85)
Biocor/Epic	24 (22-26)	56 (53-58)	80 (76-84)
Mitroflow/Crown	27 (24-29)	70 (66-74)	91 (87-95)
Soprano	23 (20-26)	53 (50-57)	77 (72-82)
Trifecta	30 (21-39)	NA	NA
**Heart failure hospitalization** ^a^			
Perimount	6.6 (6.1-7.2)	14.0 (13.1-14.9)	20.9 (19.2-22.7)
Mosaic/Hancock	8.3 (6.8-9.9)	16.0 (13.6-18.3)	22.6 (19.4-25.7)
Biocor/Epic	10.4 (8.9-11.8)	18.9 (16.9-20.9)	25.0 (21.7-28.2)
Mitroflow/Crown	9.6 (7.9-11.2)	25.9 (22.3-29.4)	28.9 (25.0-32.8)
Soprano	8.9 (7.1-10.7)	17.3 (14.8-19.8)	NA
Trifecta	5.5 (1.2-9.8)	NA	NA

Abbreviation: NA, not available.

^a^ Reintervention and rehospitalization were investigated using the Aalen-Johansen estimator and accounted for the competing risk of death.

^b^ The cumulative incidence of death was estimated using Kaplan-Meier methods.

**Figure 1.**
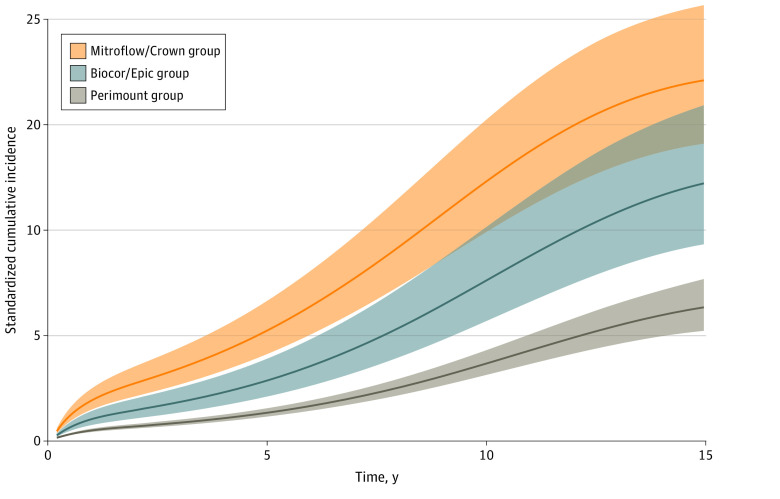
Regression-Standardized Cumulative Incidence of Reintervention, With Death as the Competing Risk The curves represent the expected outcome if the entire population received each valve model. For example, if the entire population received a Perimount valve, 3.6% of them are expected to have a reintervention in 10 years.

The adjusted cumulative incidence rates at 5, 10, and 15 years are shown in eTable 3 in the Supplement. After regression standardization, the lowest estimated cumulative incidence of reintervention was observed in the Perimount valve model group. The Soprano and the Mitroflow/Crown valve model groups had the highest cumulative incidence of reintervention, with a significant difference compared with the Perimount valve model group throughout the study period. The estimated cumulative incidence rates at 10 years were 3.6% (95% CI, 3.1%-4.2%) in the Perimount, 11.7% (95% CI, 9.2%-14.8%) in the Soprano, and 12.2% (95% CI, 9.8%-15.1%) in the Mitroflow/Crown valve model groups. The regression-standardized cumulative incidence of reintervention with death as a competing risk for the Perimount, Biocor/Epic, and Mitroflow/Crown valve model groups is shown in Figure 1. The regression-standardized cumulative incidence of reintervention with death as a competing risk for all model groups is shown in eFigure 5 in the Supplement.

### All-Cause Mortality

During a follow-up time of 106 661 person-years (mean follow-up: 6.3 years; maximum follow-up: 17.2 years), 6731 patients died. Crude and age- and sex-adjusted incidence rates are presented in the eResults and eTable 2 in the Supplement. The crude cumulative incidence of death at 10 years was lowest in the Perimount valve model group (44%; 95% CI, 43%-45%). The Mitroflow/Crown valve model group had the highest crude cumulative incidence of death at 10 years (70%; 95% CI, 66%-74%) (Table 2). The estimated survival rates in the Perimount valve model group, using the Kaplan-Meier method, were 82% (95% CI, 81%-82%) at 5 years, 56% (95% CI, 55%-57%) at 10 years, and 28% (95% CI, 25%-30%) at 15 years.

After regression standardization, the estimated survival rate was highest in the Perimount, Mosaic/Hancock, and Biocor/Epic valve model groups. However, the Mitroflow/Crown valve model group had the lowest survival rate, with a significant difference throughout the study period. The estimated cumulative incidence of all-cause mortality at 10 years was 44% (95% CI, 43%-45%) in the Perimount and 54% (95% CI, 52%-57%) in the Mitroflow/Crown valve model groups (eTable 3 in the Supplement). In the Mitroflow/Crown valve model group, after regression standardization, the 10-year survival rate was 46% (95% CI, 43%-48%) and the estimated freedom from reintervention was 88% (95% CI, 85%-90%). At 5 years, the crude 5-year survival rate was 77% (95% CI, 75%-80%) and the adjusted rate was 79% (95% CI, 77%-81%) for the Soprano valve model group.

**Figure 2.**
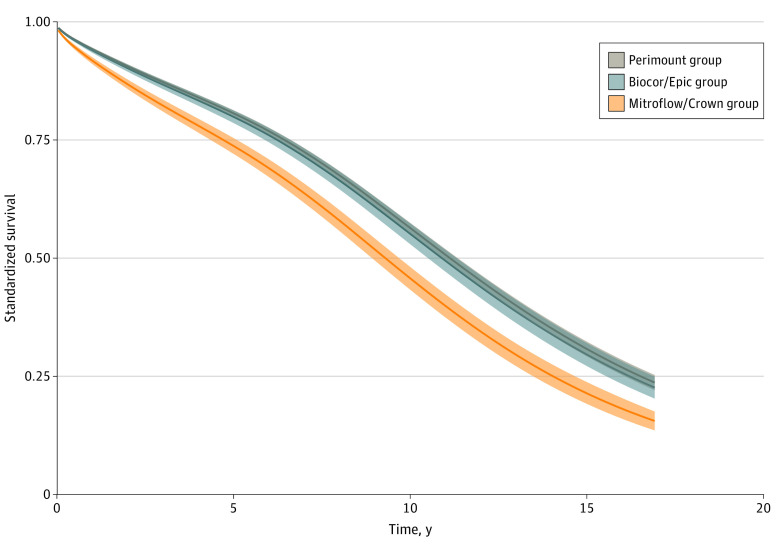
Regression-Standardized Survival The curves represent the expected survival if the entire population received each valve model. For example, if the entire population received a Perimount valve, 56% of them are expected to be alive in 10 years.

Regression-standardized survival curves for the Perimount, Biocor/Epic, and Mitroflow/Crown valve model groups are shown in Figure 2. Regression-standardized survival curves for all groups are shown in eFigure 6 in the Supplement.

### Heart Failure Hospitalization

During a follow-up time of 112 671 person-years (mean follow-up: 6.3 years; maximum follow-up: 16.0 years), 1950 patients were hospitalized for heart failure. Crude and age- and sex-adjusted incidence rates are presented in the eResults and eTable 2 in the Supplement. The Perimount valve model group had the lowest crude cumulative incidence rate at 10 years (14.0%; 95% CI, 13.1%-14.9%), and the highest rate was observed in the Mitroflow/Crown valve model group (25.9%; 95% CI, 22.3%-29.4%) (Table 2).

After regression standardization, the Perimount valve model group had the lowest estimated cumulative incidence of heart failure hospitalization. The Mitroflow/Crown valve model group had the highest estimated cumulative incidence, with a significant difference throughout the study period. The estimated cumulative incidence rates at 10 years were 12.9% (95% CI, 12.0%-13.8%) in the Perimount and 19.9% (95% CI, 17.6%-22.5%) in the Mitroflow/Crown valve model groups (eTable 3 in the Supplement).

**Figure 3.**
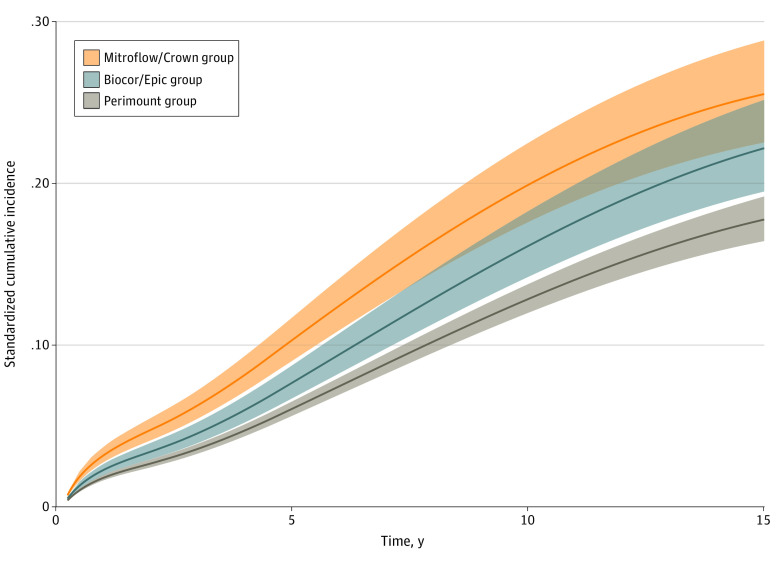
Regression-Standardized Cumulative Incidence of Heart Failure Hospitalization, With Death as the Competing Risk The curves represent the expected outcome if the entire population received each valve model. For example, if the entire population received a Perimount valve, 12.9% of them are expected to be hospitalized for heart failure in 10 years.

The regression-standardized cumulative incidence of heart failure hospitalization with death as a competing risk for the Mitroflow/Crown, Biocor/Epic, and Perimount valve model groups is shown in Figure 3. The regression-standardized cumulative incidence of heart failure hospitalization with death as a competing risk for all groups is shown in eFigure 7 in the Supplement.

### Sensitivity Analyses

We found that the results of the sensitivity analyses were similar to the results of the main analyses. On a model level, there were small and nonsignificant differences between the valves in each model group. The estimated difference in cumulative incidence of reintervention at 10 years between Biocor and Epic valves was 2% (6.3% [95% CI, 3.9%-10%] vs 8.3% [95% CI, 5.9%-11.6%]).

The estimated difference in cumulative incidence of reintervention at 10 years between Hancock II and Mosaic valves was 1.5% (4.6% [95% CI, 2.7%-7.8%] vs 6.1% [95% CI, 4.0%-9.3%]). The estimated difference in cumulative incidence of all-cause mortality at 10 years between Biocor and Epic valves was 2% (46% [95% CI, 42%-50%] vs 44% [95% CI, 42%-47%]). The estimated difference in cumulative incidence of all-cause mortality at 10 years between Hancock II and Mosaic valves was 1% (44% [95% CI, 40%-47%] vs 45% [95% CI, 42%-48%]). Results on model level are presented in eTables 5 to 9 and eFigures 8 to 13 in the Supplement.

## Discussion

We found that patients in the Perimount valve model group had lower rates of reintervention, all-cause mortality, and heart failure hospitalization compared with patients in the other valve model groups. Patients in the Mitroflow/Crown and Soprano valve model groups had the highest estimated incidence of reintervention. Patients in the Mitroflow/Crown valve model group also had the highest estimated risk of all-cause mortality and heart failure hospitalization. These results were in line with findings from the study by Hickey et al,^15^ who analyzed the performance of prosthetic valves used in the United Kingdom. Hickey et al^15^ identified 2 bioprosthetic valve series (Mitroflow and Sorin biological valves, of which 39% was Soprano) that had a higher association with a composite end point of reintervention and death compared with the other valve series. The authors reported a cumulative freedom from the composite end point at 10 years of 33.8% for Mitroflow valves and 41.4% for Sorin biological valves. Three valve series (Hancock, Perimount Magna, and Perimount) had a lower association with the composite end point compared with the other valve series. A major strength of the study by Hickey et al^15^ was its large study population (n = 54 866). This strength enabled the detailed analysis of valve models, whereas the present study needed to group models more inclusively.

In this cohort study, additional sensitivity analyses on model level showed that the differences between the valve models in each group were small and not clinically relevant. Although these findings support the rationale to analyze valve model groups in this study, heart valve upgrades or modifications within the same series cannot be assumed to yield similar or improved results as compared with the previous model.

The Hickey et al^15^ choice of shared frailties as the effect measure provided an intuitive interpretation; more than 1 indicated an increased risk of the outcome and less than 1 indicated a decreased risk of the outcome. However, we used regression standardization, which provided additional information while retaining an intuitive interpretation. For example, in assessing the clinical significance of the difference in reintervention between Mitroflow and Perimount valves, regression standardization allowed us to estimate this difference as 8.6% after 10 years, whereas a shared frailty of 1.2 compared with 0.9 was not as helpful.

The Perimount valve is one of the most frequently used and well-studied valves in the Western world.^34^ Early generations of this valve have been in clinical use since the 1980s, and subsequent generations have the same general design but with small modifications to ease supra-annular placement and to improve tissue preservation.^35^ Hemodynamic performance and long-term follow-up of the Perimount valve have provided satisfactory results.^12,13,34,36,37,38^ A recent study by Lam and colleagues^34^ reported an 8-year cumulative incidence of reintervention of 0.7% compared with a 10-year cumulative incidence of reintervention of 3% in the current study. In a recent study by Krasniqi et al,^12^ which investigated the long-term performance of Perimount valves in Denmark, the cumulative incidence rates of reintervention were 1.3% at 5 years, 5.0% at 10 years, and 13.3% at 15 years. Although the cumulative incidence rates of reintervention at 5 and 10 years were similar to the rates in the present study, the 15-year estimate was much higher in this Danish cohort (13.3% vs 5.1%). This difference between studies might be explained in part by a smaller sample size or variation in population. In the Danish cohort, the Kaplan-Meier estimated survival rates were 77% at 5 years, 52% at 10 years, and 24% at 15 years,^12^ which are similar to those found in the current study (82% at 5 years, 56% at 10 years, and 28% at 15 years).

The literature regarding the long-term performance of the Soprano valve is sparse. Early and mid-term studies have reported a 5-year survival of 81% for this valve.^39,40^ In the present study, the crude 5-year survival rate was estimated at 77% and the adjusted rate was 79%, which were similar to the corresponding estimates for other valve model groups. We observed an increased risk for reintervention with the Soprano valve model group compared with the other valve model groups, which, to our knowledge, has not been reported to date.

The Trifecta valve is a bovine, externally wrapped bioprosthesis. A study by Yongue et al^41^ compared 2305 patients who received a Trifecta prosthesis to 17 281 matched patients who received a Perimount prosthesis. Yongue et al^41^ found that the Trifecta valve was associated with superior early hemodynamic performance but with a lower freedom from explant at 5 years compared with the Perimount valve. In the present study, the findings regarding the Trifecta valve model group should be interpreted with caution owing to the small number of patients in this group.

Previous studies on the Mitroflow valve have reported varying results.^9^ The early generation of this valve was prone to valve failure owing to abrasions of the leaflets from the ribbed Dacron stent coating. This problem was rectified in a subsequent model (model 12A/LX). However, this model did not have any anticalcification treatment, and concern was raised regarding potential early valve calcification.^42^ The next model (DL) included anticalcification treatment, but it also had an accelerated incidence of failure.^43,44^ A new model, with a different tissue preservation and an anticalcification method but otherwise similar construction, was introduced as the Crown PRT after receiving European Conformitè Europëenne approval in 2014. Long-term results for the Crown model are lacking. In a large, multicenter study that investigated the long-term performance of the Mitroflow prosthesis using a single valve series, the 10-year actuarial freedom of death rate was 34.4% and reoperation rate was 67.1%.^45^ This previous study concluded that the Mitroflow valve provided beneficial valve durability and clinical outcomes.^45^ These rates correspond to the estimates in the present study of 46% for 10-year survival and 88% for freedom from reintervention in the Mitroflow/Crown valve model group. Although the estimates in the present study appear to be more favorable, our conclusions regarding the long-term performance of Mitroflow/Crown valves are different from those of the previous study. This discrepancy indicates the potential pitfalls in assessing the performance of prosthetic valves without conducting a direct comparison to the same population.

Patients are likely to be more interested in knowing which valve model among all of the available prostheses would maximize survival and minimize the risk of having to repeat the procedure. By using regression standardization and comparing multiple valve models from the same population, we provided clinically relevant information to answer this question.

### Strengths and Limitations

This study has some strengths. First, we used large, nationwide registries with near-complete follow-up data. Follow-up on mortality was complete, and because of universal health care coverage in Sweden, we believe most Swedish citizens who need reintervention would choose to have the procedure performed in Sweden. Therefore, these patients would be included in the national registries. However, we would not have access to the data of patients who choose to have a reintervention in other countries. Second, there was only a small amount of missing data. Third, we used regression standardization, which enabled us to represent the population implications of the valve model group for the various outcomes in an intuitive and graphical manner.

This study also has several limitations. First, because of the limited number of patients in the Trifecta valve model group, the results for this group should be interpreted with caution. In addition, a larger study population would have allowed us to perform the main analyses on model level rather than on model group level. Second, echocardiographic data were not available in the Swedish national health data registers. Therefore, we were unable to obtain these data for the study population. Consequently, we were unable to study the association of valve model with structural valve disease according to current definitions other than in terms of its clinical manifestation of reintervention, mortality, or heart failure.^46^ Moreover, manufacturer-supplied valve sizes are not directly comparable across valve models, and gradients may differ between valves of the same size but of different models. Third, we lacked data on the operating surgeon. However, we had access to data on the surgical center, which was a covariate in all models. The SWEDEHEART register contains transparent and open reporting of cardiac surgery volumes and quality of care in Sweden, and similar results have been reported throughout the years.

## Conclusions

The Perimount valve model group had the lowest rates of reintervention, all-cause mortality, and heart failure hospitalization, and it was the most commonly used valve type during the study period. The Mitroflow/Crown valve model group had high rates of reintervention, all-cause mortality, and heart failure hospitalization, which were a deviation in performance. The Soprano valve model group also had an increased incidence of reintervention. These findings support the extensive use of the Perimount valve and suggest the need for increased clinical vigilance in patients who receive either a Soprano valve or a Mitroflow/Crown valve.

## Notes

Supplement. **eMethods.** Statistical Methods
eReferences
**eResults.** Supplemental Results **eTable 1.** Frequency of Valve Models per Model Group **eTable 2.** Incidence Rates of Reintervention, All-Cause Mortality and Heart Failure Hospitalization Following Bioprosthetic Aortic Valve Replacement in Sweden Between 2003 and 2018 **eTable 3.** Adjusted Cumulative Incidence for Reintervention, All-Cause Mortality and Heart Failure Hospitalization at 5, 10 and 15 Years Following Bioprosthetic Aortic Valve Replacement in Sweden Between 2003 and 2018 **eTable 4.** Baseline Characteristics of 16,983 Patients Who Underwent Bioprosthetic Aortic Valve Replacement in Sweden Between 2003 and 2018, Stratified by Outcome **eFigure 1.** Valve Model Group by Year **eFigure 2.** Age Distribution by Model Group **eFigure 3.** Valve Size Distribution by Model Group **eFigure 4.** LVEF Distribution by Model Group **eFigure 5.** Regression Standardized Cumulative Incidence of Reintervention, Accounting for the Competing Risk of Death **eFigure 6.** Regression Standardized Survival **eFigure 7.** Regression Standardized Cumulative Incidence of Heart Failure Hospitalization Accounting for the Competing Risk of Death **eTable 5.** Baseline Characteristics of 16,983 Patients Who Underwent Bioprosthetic Aortic Valve Replacement in Sweden Between 2003 and 2018, Stratified by Model **eTable 6.** Baseline Characteristics of 16,983 Patients Who Underwent Bioprosthetic Aortic Valve Replacement in Sweden Between 2003 and 2018, Stratified by Outcome **eTable 7.** Crude Cumulative Incidence of Reintervention, All-Cause Mortality and Heart Failure Hospitalization at 5, 10 and 15 Years Following Bioprosthetic Aortic Valve Replacement in Sweden Between 2003 and 2018, per Model **eTable 8.** Incidence Rates of Reintervention, All-Cause Mortality and Heart Failure Hospitalization Following Bioprosthetic Aortic Valve Replacement in Sweden Between 2003 and 2018, Stratified by Model **eTable 9.** Adjusted Cumulative Incidence for Reintervention, All-Cause Mortality and Heart Failure Hospitalization at 5, 10 and 15 Years Following Bioprosthetic Aortic Valve Replacement in Sweden Between 2003 and 2018, Stratified by Model **eFigure 8.** Regression Standardized Cumulative Incidence of Reintervention, Accounting for the Competing Risk of Death **eFigure 9.** Regression Standardized Cumulative Incidence of Reintervention, Accounting for the Competing Risk of Death, All Models **eFigure 10.** Regression Standardized Survival **eFigure 11.** Regression Standardized Survival, All Models **eFigure 12.** Regression Standardized Cumulative Incidence of Heart Failure Hospitalization, Accounting for the Competing Risk of Death **eFigure 13.** Regression Standardized Cumulative Incidence of Heart Failure Hospitalization, Accounting for the Competing Risk of Death, All Models
