# Supplementary material for: Comparison of Long-term Performance of Bioprosthetic Aortic Valves in Sweden From 2003 to 2018
Source: JAMA Netw Open. 2022 Mar 7;5(3):e220962. doi: 10.1001/jamanetworkopen.2022.0962 (PMC8902647; doi:10.1001/jamanetworkopen.2022.0962)
Supplement: Supplement. — eMethods. Statistical Methods eReferences eResults. Supplemental Results eTable 1. Frequency of Valve Models per Model Group eTable 2. Incidence Rates of Reintervention, All-Cause Mortality and Heart Failure Hospitalization Following Bioprosthetic Aortic Valve Replacement in Sweden Between 2003 and 2018 eTable 3. Adjusted Cumulative Incidence for Reintervention, All-Cause Mortality and Heart Failure Hospitalization at 5, 10 and 15 Years Following Bioprosthetic Aortic Valve Replacement in Sweden Between 2003 and 2018 eTable 4. Baseline Characteristics of 16,983 Patients Who Underwent Bioprosthetic Aortic Valve Replacement in Sweden Between 2003 and 2018, Stratified by Outcome eFigure 1. Valve Model Group by Year eFigure 2. Age Distribution by Model Group eFigure 3. Valve Size Distribution by Model Group eFigure 4. LVEF Distribution by Model Group eFigure 5. Regression Standardized Cumulative Incidence of Reintervention, Accounting for the Competing Risk of Death eFigure 6. Regression Standardized Survival eFigure 7. Regression Standardized Cumulative Incidence of Heart Failure Hospitalization Accounting for the Competing Risk of Death eTable 5. Baseline Characteristics of 16,983 Patients Who Underwent Bioprosthetic Aortic Valve Replacement in Sweden Between 2003 and 2018, Stratified by Model eTable 6. Baseline Characteristics of 16,983 Patients Who Underwent Bioprosthetic Aortic Valve Replacement in Sweden Between 2003 and 2018, Stratified by Outcome eTable 7. Crude Cumulative Incidence of Reintervention, All-Cause Mortality and Heart Failure Hospitalization at 5, 10 and 15 Years Following Bioprosthetic Aortic Valve Replacement in Sweden Between 2003 and 2018, per Model eTable 8. Incidence Rates of Reintervention, All-Cause Mortality and Heart Failure Hospitalization Following Bioprosthetic Aortic Valve Replacement in Sweden Between 2003 and 2018, Stratified by Model eTable 9. Adjusted Cumulative Incidence for Reintervention, All-Cause Mortality and Heart Failure Hos [file jamanetwopen-e220962-s001.pdf]

Supplementary Online Content

Persson M, Glaser N, Nilsson J, Friberg Ö, Franco-Cereceda A, Sartipy U. Comparison of long-term performance of bioprosthetic aortic valves in Sweden from 2003 to 2018. *JAMA Netw Open*. 2022;5(3):e220962. doi:10.1001/jamanetworkopen.2022.0962

eMethods. Statistical Methods
eReferences
eResults. Supplemental Results
eTable 1. Frequency of Valve Models per Model Group
eTable 2. Incidence Rates of Reintervention, All-Cause Mortality and Heart Failure Hospitalization Following Bioprosthetic Aortic Valve Replacement in Sweden Between 2003 and 2018
eTable 3. Adjusted Cumulative Incidence for Reintervention, All-Cause Mortality and Heart Failure Hospitalization at 5, 10 and 15 Years Following Bioprosthetic Aortic Valve Replacement in Sweden Between 2003 and 2018
eTable 4. Baseline Characteristics of 16,983 Patients Who Underwent Bioprosthetic Aortic Valve Replacement in Sweden Between 2003 and 2018, Stratified by Outcome
eFigure 1. Valve Model Group by Year
eFigure 2. Age Distribution by Model Group
eFigure 3. Valve Size Distribution by Model Group
eFigure 4. LVEF Distribution by Model Group
eFigure 5. Regression Standardized Cumulative Incidence of Reintervention, Accounting for the Competing Risk of Death
eFigure 6. Regression Standardized Survival
eFigure 7. Regression Standardized Cumulative Incidence of Heart Failure Hospitalization Accounting for the Competing Risk of Death
eTable 5. Baseline Characteristics of 16,983 Patients Who Underwent Bioprosthetic Aortic Valve Replacement in Sweden Between 2003 and 2018, Stratified by Model
eTable 6. Baseline Characteristics of 16,983 Patients Who Underwent Bioprosthetic Aortic Valve Replacement in Sweden Between 2003 and 2018, Stratified by Outcome
eTable 7. Crude Cumulative Incidence of Reintervention, All-Cause Mortality and Heart Failure Hospitalization at 5, 10 and 15 Years Following Bioprosthetic Aortic Valve Replacement in Sweden Between 2003 and 2018, per Model
eTable 8. Incidence Rates of Reintervention, All-Cause Mortality and Heart Failure Hospitalization Following Bioprosthetic Aortic Valve Replacement in Sweden Between 2003 and 2018, Stratified by Model
eTable 9. Adjusted Cumulative Incidence for Reintervention, All-Cause Mortality and Heart Failure Hospitalization at 5, 10 and 15 Years Following Bioprosthetic Aortic Valve Replacement in Sweden Between 2003 and 2018, Stratified by Model
eFigure 8. Regression Standardized Cumulative Incidence of Reintervention, Accounting for the Competing Risk of Death
eFigure 9. Regression Standardized Cumulative Incidence of Reintervention, Accounting for the Competing Risk of Death, All Models

© 2022 Persson M et al. *JAMA Network Open.*

eFigure 10. Regression Standardized Survival
eFigure 11. Regression Standardized Survival, All Models
eFigure 12. Regression Standardized Cumulative Incidence of Heart Failure Hospitalization, Accounting for the Competing Risk of Death
eFigure 13. Regression Standardized Cumulative Incidence of Heart Failure Hospitalization, Accounting for the Competing Risk of Death, All Models

This supplementary material has been provided by the authors to give readers additional information about their work.

© 2022 Persson M et al. *JAMA Network Open.*

eMethods: Statistical Methods

Regression standardization

In biostatistics, regression models are frequently used to study the association between exposure and outcome while adjusting for potential confounders, which are introduced as covariates in the regression model. An example of this regression is that when studying binary outcomes, logistic regression is frequently used to obtain odds ratios for exposure versus nonexposure for the outcome in question. The predicted odds for a particular patient can also be estimated by simply providing the model with that patient's values for the different covariates (e.g. age = 50 years, sex = male, diabetes mellitus = false and exposure = true). In survival or time-to-event analysis, one of the most frequently used regression models is the Cox proportional hazards model. This method is used to obtain hazard ratios for exposure versus non-exposure for the outcome in question. Similarly, as in the example above, the resulting model can be used to predict a particular patient's hazard for the outcome by providing that patient's covariate pattern in the model. These estimates can be used to draw an adjusted survival curve that accounts for potential confounders of that patient. Frequently, this is not what the researcher wishes to achieve, but rather the researcher wishes to draw an adjusted survival curve for the population, similar to the unadjusted Kaplan-Meier curve. One way to acheive this goal is to simply impute the population mean for the covariates. However, this imputation produces an estimate for a hypothetical patient, potentially with age = 74 years, sex = 0.5, treatment = 0.7. Obviously, this result is not interpretable because one can only be one of a binary covariate, i.e. sex can only be 0 or 1 (male or female) and not 0.5. Likewise, treatment can only be 0 or 1 (untreated or treated), not 0.7. An alternative is to use the population mean for continuous covariates and the mode for categorical variables. However, this also produces an estimate for a hypothetical patient (e.g., with a covariate pattern of age = 74 years, sex = male, and treatment = 0). This result might be acceptable if only few binary covariates are used, and then one curve for men and one curve for women might be drawn for each of the exposure levels. However, this is frequently not the case, but rather, a model may have 15 covariates, with

© 2022 Persson M et al. *JAMA Network Open.*

some that are some continuous, some that are categorical, and some that are binary. One solution for this problem is regression standardization. In survival analysis, this helps researchers obtain a direct standardized survival curve. The method for this analysis is as follows. First, the regression model is run with the exposure, outcome and potential confounders as covariates. The exposure is then recoded for all patients in the population to true. This model is then used to predict the effect measure for each of the patients in the population, and finally, the average these predictions. This procedure is repeated, but the exposure is set to the next level (or in this example with a binary exposure, the exposure is set to false). In this example, the method will provide two survival curves of one for exposed, and one for non-exposed. Each curve represents the hypothetical population where all patients either received or did not receive the treatment, standardized by (i.e., adjusted for) the population distribution of covariates. The results are intuitive and can be interpreted as follows. If the entire population received treatment X, 70% would be alive at 10 years. If the entire population received treatment Y instead, only 50% would be alive at 10 years". Regression standardization answers what in causal inference often is referred to as a counterfactual question, characterized by its "if instead…" nature. In fact, if measured covariates are sufficient for confounder control, these results can be interpreted as causal effects for the population under study. For further reading, we recommend Sjölander 2016, Kipouro 2019 and Rothman 2021 p.475-478, 518-521.[1–3]

Model selection

We used a model selection strategy that combined subject matter knowledge and backward selection using all variables in Table 1 with the addition of surgical center. The final model selection was informed using the Akaike information criterion (AIC).

Reintervention

For reintervention, the final model used a baseline hazard described by a third degree exponential B-spline and two interior knots placed at the first and second tertiles of the follow-up time. Included covariates were as follows: model group, sex, surgical center, concomitant

© 2022 Persson M et al. *JAMA Network Open.*

coronary bypass grafting (CABG), concomitant ascending aortic surgery, prior atrial fibrillation, prior myocardial infarction, prior endocarditis, hyperlipidemia, prior pacemaker (PM)/implantable cardioverter defibrillator (ICD), body mass index (BMI) category, period of surgery, estimated glomerular filtration rate (eGFR) category, valve size category, and age. Using the AIC, age was centered and included as a quadratic term.

All-cause mortality in the competing risk setting

For all-cause mortality in the competing risk setting, the baseline hazard was modeled as above. Included covariates were as follows: model group, sex, surgical center, left ventricular ejection fraction (LVEF) category, emergent operation, concomitant CABG, birth region, education level, prior atrial fibrillation, alcohol dependency, prior myocardial infarction, history of cancer, prior chronic obstructive pulmonary disease (COPD), prior diabetes mellitus (DM), prior endocarditis, prior heart failure, hyperlipidemia, hypertension, prior hepatic disease, prior peripheral vascular disease, prior stroke, prior PM/ICD, prior percutaneous coronary intervention (PCI), marital status, disposable income category, BMI category, period of surgery, eGFR category, valve size category, and age. Using the AIC, age was centered and included as a time varying covariate and a quadratic term.

Heart failure hospitalization

For heart failure hospitalization, the baseline hazard was modeled as for reintervention above. Included covariates were as follows: model group, sex, surgical center, LVEF category, concomitant CABG, concomitant ascending aortic surgery, birth region, education level, prior atrial fibrillation, alcohol dependency, prior myocardial infarction, history of cancer, prior COPD, prior DM, hypertension, prior peripheral vascular disease, prior PM/ICD, prior PCI, disposable income category, BMI category, period of surgery, eGFR category, valve size category and age. Using the AIC, age was centered and included as a quadratic term.

All-cause mortality

For all-cause mortality in survival analysis, the baseline hazard was modeled using a natural spline with three degrees of freedom. Included variables were as follows: model group, sex,

© 2022 Persson M et al. *JAMA Network Open.*

surgical center, LVEF category, emergent operation, concomitant CABG, concomitant ascending aortic surgery, birth region, education level, prior atrial fibrillation, alcohol dependency, prior myocardial infarction, history of cancer, prior COPD, prior DM, prior endocarditis, prior heart failure, hyperlipidemia, hypertension, prior hepatic disease, prior peripheral vascular disease, prior stroke, prior PM/ICD, prior PCI, marital status, disposable income category, BMI category, period of surgery, eGFR category, valve size category and age. Using the AIC, age was centered and included as a cubic spline and as a time varying covariate.

© 2022 Persson M et al. *JAMA Network Open.*

eReferences

1. Sjölander A. Regression standardization with the R package stdReg. *Eur J Epidemiol*. 2016;31(6):563-574. doi:10.1007/s10654-016-0157-3

2. Kipourou D-K, Charvat H, Rachet B, Belot A. Estimation of the adjusted cause-specific cumulative probability using flexible regression models for the cause-specific hazards. *Stat Med*. 2019;38(20):3896-3910. doi:10.1002/sim.8209

3. Rothman KJ, Lash TL, VanderWeele TJ, Haneuse S. *Modern Epidemiology*. Fourth edition. Wolters Kluwer; 2021.

© 2022 Persson M et al. *JAMA Network Open.*

eResults: Supplemental Results

Crude and age- and sex-adjusted incidence rates

Reintervention

Crude and age- and sex-adjusted incidence rates are reported in eTable 2. The Perimount group had the lowest crude and age- and sex-adjusted incidence rates of 0.34% (95% CI 0.30%-0-38%) and 0.32% (95% CI 0.25%-0.40%), respectively. These rates were similar to the Mosaic/Hancock group, with an age- and sex-adjusted incidence rate of 0.52% (95% CI 0.36%-0.74%). The Trifecta group had the highest crude, and age- and sex-adjusted incidence rates of 1.02% (95% CI 0.41%-2.10%) and 2.24% (95% CI 0.77%-6.51%), respectively. The second highest rates were found in the Soprano group in crude analysis (0.91%, 95% CI 0.73%-1.13%) and in the Mitroflow/Crown group in age- and sex-adjusted analysis (1.35%, 95% CI 0.97%-1.87%).

All-cause mortality

The Perimount group had the lowest crude (5.5%, 95% CI 5.3%-5.6%) and age- and sex-adjusted (6.0%, 95% CI 5.6%-6.4%) incidence rates of death. The Mitroflow/Crown group had the highest crude (8.4%, 95% CI 7.8%-9.1%) and age- and sex-adjusted (7.9%, 95% CI 7.4%-8.4%) incidence rates of death (eTable 2).

Heart failure hospitalization

The Perimount group had the lowest crude and age- and sex-adjusted incidence rates of 1.50% (95% CI 1.41%-1.59%) and 1.54% (95% CI 1.35%-1.77%), respectively, for heart failure hospitalization. The Mitroflow/Crown group had the highest crude and age- and sex-adjusted incidence rates of 2.62% (95% CI 2.29%-3.00%) and 2.46% (95% CI 2.13%-2.85%) respectively (eTable 2).

© 2022 Persson M et al. *JAMA Network Open.*

eTable 1. Frequency of Valve Models per Model Group

	No.
Perimount	
Carpentier-Edwards Unknown	38
Perimount 2900	8801
Perimount SERIALNO	430
Perimount Magna 3000	346
Perimount Magna Ease 3300TFX	1644
Perimount Unknown	10
Mosaic/Hancock	
Mosaic	446
Hancock or Mosaic SERIALNO	91
Hancock II Aorta Ultra T505U	72
Hancock II Aorta T505C	622
Mosaic Aortic Ultra	4
Epic/Biocor	
Biocor	419
St. Jude Epic	244
St. Jude Epic Aortic Valve EL-##A	93
St. Jude Epic Supra Aortic	64
Epic Valve Aortic E100-**/A	725
Epic Supravalve Aortic ESP100-**/A	125
Mitroflow/Crown	
Mitroflow Aortic	1064
Crown PRT	579
Soprano	
Soprano Armonia	602
Soprano Armonia SOP	372
Trifecta	
Trifecta	192

© 2022 Persson M et al. *JAMA Network Open.*

eTable 2. Incidence Rates of reintervention, all-cause mortality and heart failure hospitalization following bioprosthetic aortic valve replacement in Sweden between 2003 and 2018

Model group	Incidence rate / 100 person-years (95% CI)		
	Reintervention	All-cause mortality	Heart failure hospitalization
Crude			
Perimount	0.34 (0.30-0.38)	5.5 (5.3-5.6)	1.50 (1.41-1.59)
Mosaic/Hancock	0.34 (0.24-0.46)	7.4 (6.9-8.0)	1.85 (1.60-2.12)
Biocor/Epic	0.37 (0.28-0.47)	7.9 (7.5-8.4)	2.12 (1.89-2.37)
Mitroflow/Crown	0.88 (0.69-1.09)	8.4 (7.8-9.1)	2.62 (2.29-3.00)
Soprano	0.91 (0.73-1.13)	7.6 (7.0-8.2)	1.91 (1.63-2.22)
Trifecta	1.02 (0.41-2.10)	7.0 (5.2-9.1)	1.85 (0.95-3.23)
Age- and sex-adjusted			
Perimount	0.32 (0.25-0.40)	6.0 (5.6-6.4)	1.54 (1.35-1.77)
Mosaic/Hancock	0.52 (0.36-0.74)	6.8 (6.5-7.2)	1.74 (1.54-1.96)
Biocor/Epic	0.55 (0.48-0.62)	7.1 (6.9-7.3)	1.92 (1.80-2.04)
Mitroflow/Crown	1.35 (0.97-1.87)	7.9 (7.4-8.4)	2.46 (2.13-2.85)
Soprano	1.08 (0.86-1.34)	7.6 (7.1-8.1)	1.87 (1.61-2.18)
Trifecta	2.24 (0.77-6.51)	7.1 (5.5-9.2)	1.81 (1.03-3.18)

Age- and sex-adjusted incidence rates were obtained from a Poisson model. CI = confidence interval.

© 2022 Persson M et al. *JAMA Network Open.*

eTable 3. Adjusted Cumulative Incidence for Reintervention, All-Cause Mortality and Heart Failure Hospitalization at 5, 10 and 15 Years Following Bioprosthetic Aortic Valve Replacement in Sweden Between 2003 and 2018

Model group	Cumulative incidence, % (95% CI)		
	5 years	10 years	15 years
Reintervention			
Perimount	1.3 (1.1-1.5)	3.6 (3.1-4.2)	6.3 (5.2-7.6)
Mosaic/Hancock	1.9 (1.4-2.7)	5.4 (3.8-7.5)	9.1 (6.6-12.5)
Biocor/Epic	2.8 (2-3.8)	7.5 (5.6-10)	12.2 (9.3-15.9)
Mitroflow/Crown	5.1 (4-6.5)	12.2 (9.8-15.1)	17.1 (14.1-20.6)
Soprano	4.6 (3.5-6.1)	11.7 (9.2-14.8)	17.3 (13.9-21.4)
Trifecta	3.8 (1.8-7.7)	9.5 (4.9-17.8)	14 (7.9-24.2)
All-cause mortality			
Perimount	19 (19-20)	44 (43-45)	69 (68-71)
Mosaic/Hancock	20 (18-21)	44 (42-47)	70 (67-72)
Biocor/Epic	20 (19-21)	45 (43-47)	70 (68-73)
Mitroflow/Crown	26 (24-28)	54 (52-57)	79 (76-81)
Soprano	21 (19-23)	47 (44-50)	72 (69-75)
Trifecta	24 (19-29)	51 (43-59)	76 (69-83)
Heart failure hospitalization			
Perimount	6 (5.6-6.5)	12.9 (12-13.8)	17.8 (16.5-19.2)
Mosaic/Hancock	6.9 (6-8)	14.7 (12.8-16.9)	20.4 (17.7-23.4)
Biocor/Epic	7.7 (6.7-8.7)	16.2 (14.2-18.3)	22.2 (19.5-25.2)
Mitroflow/Crown	10.3 (9-11.7)	19.9 (17.6-22.5)	25.6 (22.6-28.9)
Soprano	7.3 (6.2-8.6)	15.2 (12.9-17.7)	20.5 (17.5-24)
Trifecta	6.6 (3.8-11.1)	13.2 (7.8-21.7)	17.3 (10.3-28.2)

Model groups were adjusted by regression standardization. A detailed description and documentation regarding included covariates are available in the Supplemental material. CI = confidence interval.

© 2022 Persson M et al. *JAMA Network Open.*

eTable 4. Baseline Characteristics of 16,983 Patients Who Underwent Bioprosthetic Aortic Valve Replacement in Sweden Between 2003 and 2018, Stratified by Outcome

Variable	Overall	No Reintervention	Reintervention	Alive	Dead	No heart failure hospitalization	Heart failure hospitalization
No.	16983	16463	520	10252	6731	15033	1950
Age, years (mean (SD))	72.6 (8.5)	72.8 (8.3)	65.1 (11.2)	70.6 (8.8)	75.7 (6.9)	72.3 (8.6)	75.3 (7.0)
Female sex	6298 (37.1)	6143 (37.3)	155 (29.8)	3608 (35.2)	2690 (40.0)	5498 (36.6)	800 (41.0)
LVEF							
<30%	888 (5.3)	861 (5.3)	27 (5.3)	412 (4.0)	476 (7.2)	771 (5.2)	117 (6.1)
30%-50%	3725 (22.1)	3632 (22.2)	93 (18.1)	1943 (19.0)	1782 (26.8)	3178 (21.3)	547 (28.4)
>50%	12225 (72.6)	11832 (72.5)	393 (76.6)	7845 (76.9)	4380 (66.0)	10961 (73.5)	1264 (65.6)
Emergent operation	289 (1.7)	278 (1.7)	11 (2.1)	129 (1.3)	160 (2.4)	265 (1.8)	24 (1.2)
Concomitant CABG	6453 (38.0)	6323 (38.4)	130 (25.0)	3386 (33.0)	3067 (45.6)	5558 (37.0)	895 (45.9)
Ascending aortic surgery	1441 (8.5)	1396 (8.5)	45 (8.7)	1088 (10.6)	353 (5.2)	1358 (9.0)	83 (4.3)
Non-Nordic birth region	1016 (6.0)	967 (5.9)	49 (9.4)	686 (6.7)	330 (4.9)	921 (6.1)	95 (4.9)
Education							
<10 years	7222 (43.0)	7032 (43.2)	190 (36.8)	3769 (37.1)	3453 (52.1)	6268 (42.2)	954 (49.8)
10-12 years	6320 (37.7)	6102 (37.5)	218 (42.2)	4058 (39.9)	2262 (34.1)	5632 (37.9)	688 (35.9)
>12 years	3241 (19.3)	3132 (19.3)	109 (21.1)	2332 (23.0)	909 (13.7)	2969 (20.0)	272 (14.2)
Prior atrial fibrillation	3202 (18.9)	3149 (19.1)	53 (10.2)	1556 (15.2)	1646 (24.5)	2639 (17.6)	563 (28.9)

© 2022 Persson M et al. *JAMA Network Open.*

Alcohol dependence	500 (2.9)	476 (2.9)	24 (4.6)	275 (2.7)	225 (3.3)	441 (2.9)	59 (3.0)
Prior myocardial infarction	2858 (16.8)	2808 (17.1)	50 (9.6)	1395 (13.6)	1463 (21.7)	2412 (16.0)	446 (22.9)
History of cancer	2911 (17.1)	2852 (17.3)	59 (11.3)	1649 (16.1)	1262 (18.7)	2584 (17.2)	327 (16.8)
COPD	1938 (11.4)	1889 (11.5)	49 (9.4)	973 (9.5)	965 (14.3)	1659 (11.0)	279 (14.3)
Diabetes mellitus	3791 (22.3)	3693 (22.4)	98 (18.8)	2027 (19.8)	1764 (26.2)	3241 (21.6)	550 (28.2)
Prior endocarditis	816 (4.8)	763 (4.6)	53 (10.2)	497 (4.8)	319 (4.7)	741 (4.9)	75 (3.8)
Prior heart failure	3701 (21.8)	3616 (22.0)	85 (16.3)	1671 (16.3)	2030 (30.2)	3175 (21.1)	526 (27.0)
Hyperlipidemia	3931 (23.1)	3810 (23.1)	121 (23.3)	2593 (25.3)	1338 (19.9)	3482 (23.2)	449 (23.0)
Hypertension	9786 (57.6)	9536 (57.9)	250 (48.1)	6089 (59.4)	3697 (54.9)	8640 (57.5)	1146 (58.8)
Hepatic disease	279 (1.6)	270 (1.6)	9 (1.7)	130 (1.3)	149 (2.2)	250 (1.7)	29 (1.5)
Peripheral vascular disease	2243 (13.2)	2193 (13.3)	50 (9.6)	1277 (12.5)	966 (14.4)	1987 (13.2)	256 (13.1)
Prior stroke	2105 (12.4)	2053 (12.5)	52 (10.0)	1147 (11.2)	958 (14.2)	1834 (12.2)	271 (13.9)
Pacemaker/ICD	467 (2.7)	463 (2.8)	4 (0.8)	229 (2.2)	238 (3.5)	388 (2.6)	79 (4.1)
Prior PCI	1561 (9.2)	1529 (9.3)	32 (6.2)	970 (9.5)	591 (8.8)	1330 (8.8)	231 (11.8)
Married	10890 (64.1)	10578 (64.3)	312 (60.0)	6714 (65.5)	4176 (62.0)	9652 (64.2)	1238 (63.5)
Model							
Perimount	11269 (66.4)	11019 (66.9)	250 (48.1)	7546 (73.6)	3723 (55.3)	10229 (68.0)	1040 (53.3)
Mosaic Hancock	1235 (7.3)	1196 (7.3)	39 (7.5)	549 (5.4)	686 (10.2)	1040 (6.9)	195 (10.0)

© 2022 Persson M et al. *JAMA Network Open.*

Biocor Epic	1670 (9.8)	1609 (9.8)	61 (11.7)	649 (6.3)	1021 (15.2)	1351 (9.0)	319 (16.4)
Mitroflow Crown	1643 (9.7)	1564 (9.5)	79 (15.2)	970 (9.5)	673 (10.0)	1425 (9.5)	218 (11.2)
Soprano	974 (5.7)	890 (5.4)	84 (16.2)	398 (3.9)	576 (8.6)	808 (5.4)	166 (8.5)
Trifecta	192 (1.1)	185 (1.1)	7 (1.3)	140 (1.4)	52 (0.8)	180 (1.2)	12 (0.6)
Household income							
Q1 (lowest)	4245 (25.0)	4125 (25.1)	120 (23.1)	1836 (17.9)	2409 (35.8)	3574 (23.8)	671 (34.4)
Q2	4245 (25.0)	4127 (25.1)	118 (22.7)	2239 (21.8)	2006 (29.8)	3695 (24.6)	550 (28.2)
Q3	4245 (25.0)	4111 (25.0)	134 (25.8)	2813 (27.4)	1432 (21.3)	3814 (25.4)	431 (22.1)
Q4 (highest)	4245 (25.0)	4097 (24.9)	148 (28.5)	3362 (32.8)	883 (13.1)	3947 (26.3)	298 (15.3)
Body mass index, kg/m^2							
<18.5	147 (0.9)	146 (1.0)	1 (0.2)	67 (0.7)	80 (1.3)	136 (1.0)	11 (0.6)
18.5-24.9	5334 (33.9)	5182 (33.9)	152 (32.5)	3060 (31.9)	2274 (37.0)	4795 (34.4)	539 (30.3)
25-29.9	6690 (42.5)	6485 (42.5)	205 (43.8)	4246 (44.3)	2444 (39.8)	5956 (42.7)	734 (41.3)
>30	3562 (22.6)	3452 (22.6)	110 (23.5)	2218 (23.1)	1344 (21.9)	3068 (22.0)	494 (27.8)
Period of surgery, years							
2003-2008	4995 (29.4)	4780 (29.0)	215 (41.3)	1473 (14.4)	3522 (52.3)	4005 (26.6)	990 (50.8)
2009-2013	6085 (35.8)	5889 (35.8)	196 (37.7)	3628 (35.4)	2457 (36.5)	5352 (35.6)	733 (37.6)
2014-2018	5903 (34.8)	5794 (35.2)	109 (21.0)	5151 (50.2)	752 (11.2)	5676 (37.8)	227 (11.6)

© 2022 Persson M et al. *JAMA Network Open.*

eGFR, mL/min/1.73 m^2							
<30	480 (2.9)	465 (2.9)	15 (2.9)	181 (1.8)	299 (4.5)	424 (2.9)	56 (2.9)
30-44	1309 (7.8)	1287 (7.9)	22 (4.3)	529 (5.2)	780 (11.8)	1086 (7.3)	223 (11.7)
45-59	3120 (18.7)	3072 (19.0)	48 (9.4)	1558 (15.4)	1562 (23.7)	2683 (18.1)	437 (23.0)
>60	11793 (70.6)	11367 (70.2)	426 (83.4)	7841 (77.6)	3952 (59.9)	10608 (71.7)	1185 (62.3)
Size, mm							
19-21	5772 (34.1)	5620 (34.3)	152 (29.3)	3158 (30.9)	2614 (39.0)	5033 (33.6)	739 (38.0)
23	6515 (38.5)	6314 (38.5)	201 (38.8)	3914 (38.3)	2601 (38.8)	5763 (38.5)	752 (38.7)
≥25	4639 (27.4)	4474 (27.3)	165 (31.9)	3152 (30.8)	1487 (22.2)	4187 (27.9)	452 (23.3)

Numbers are No. (%) unless otherwise noted. SD = standard deviation, Q = quartile, LVEF = left ventricular ejection fraction, ICD = implantable cardioverter defibrillator, PCI = percutaneous coronary intervention, eGFR = estimated glomerular filtration rate, COPD = chronic obstructive pulmonary disease, CABG = coronary artery bypass grafting

© 2022 Persson M et al. *JAMA Network Open.*

eFigure 1. Valve Model Group by Year

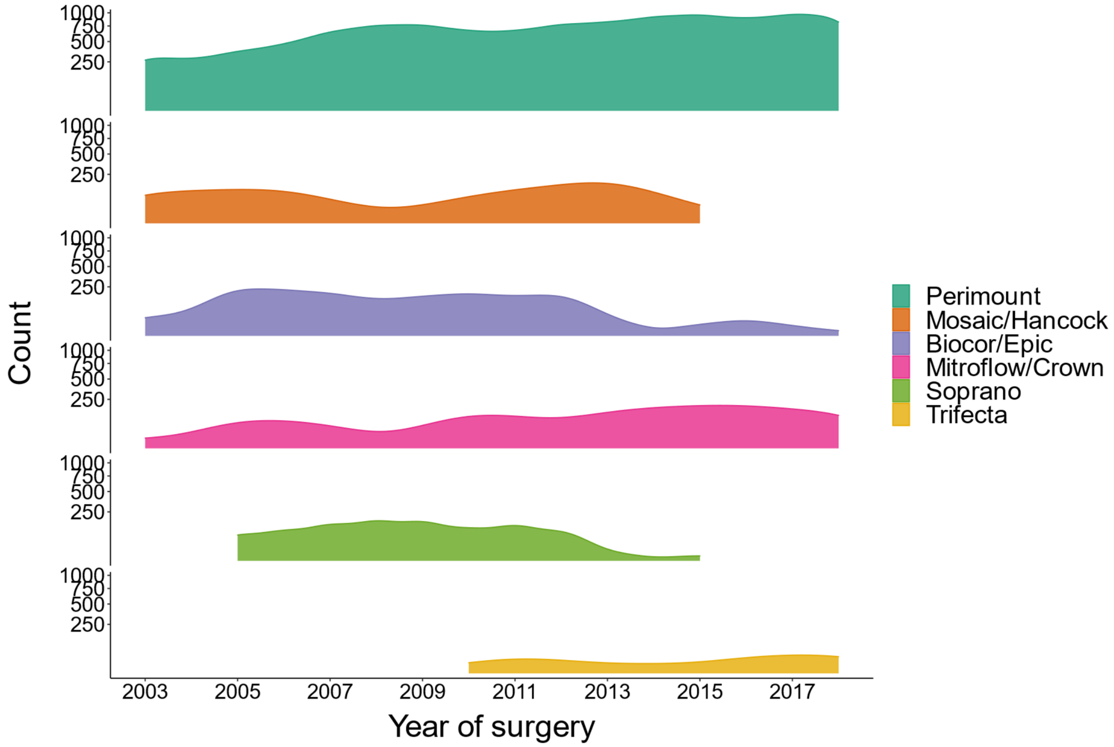

© 2022 Persson M et al. *JAMA Network Open.*

eFigure 2. Age Distribution by Model Group

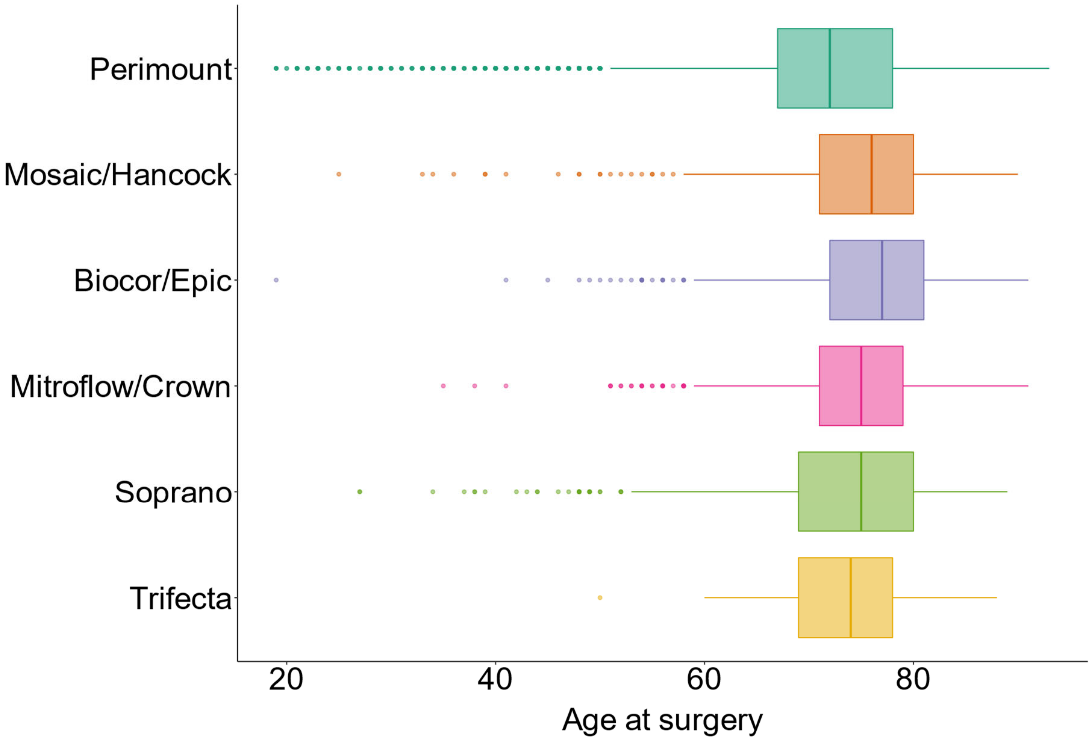

© 2022 Persson M et al. *JAMA Network Open.*

eFigure 3. Valve Size Distribution by Model Group

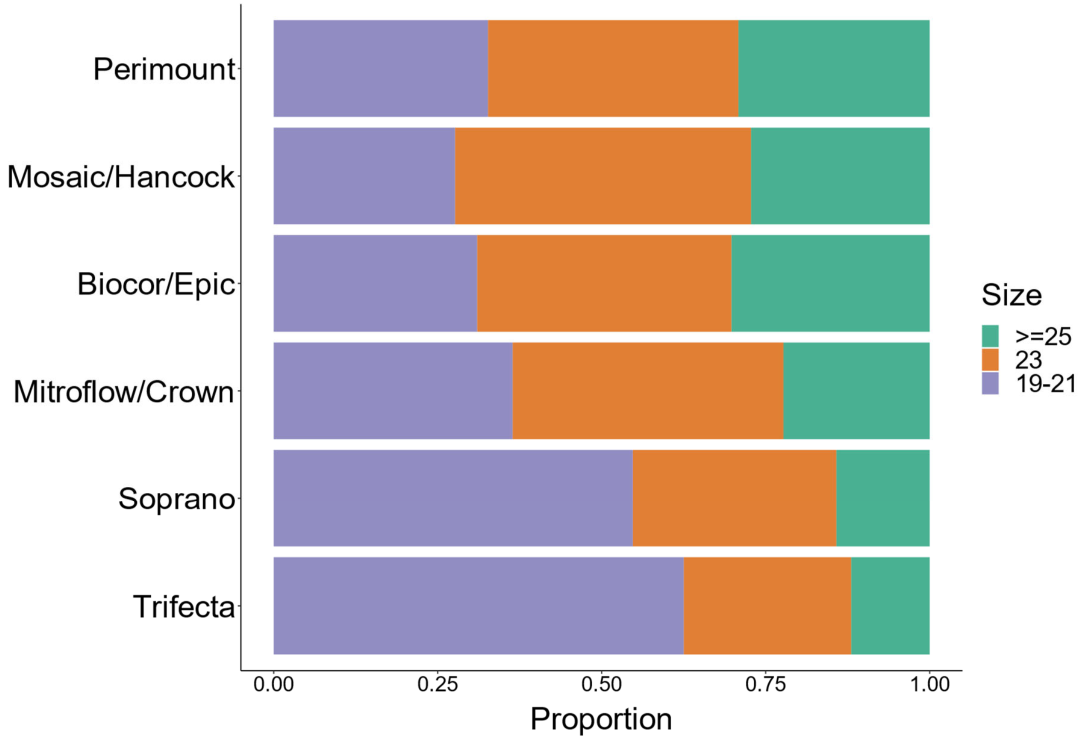

© 2022 Persson M et al. *JAMA Network Open.*

eFigure 4. LVEF Distribution by Model Group

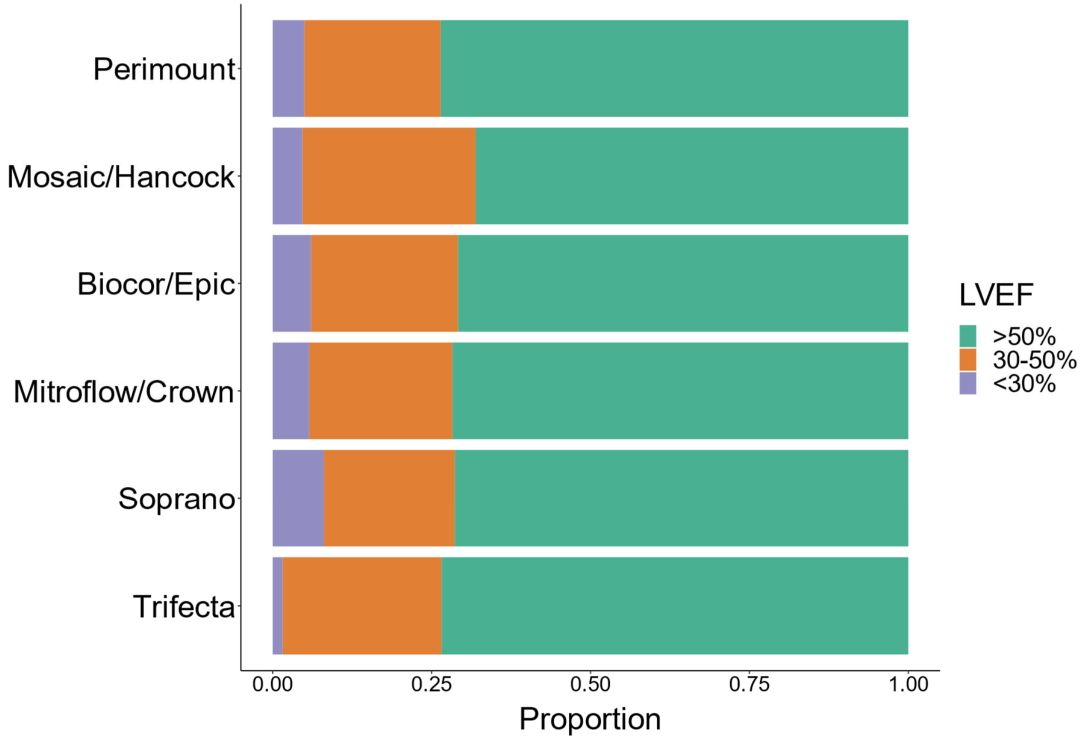

© 2022 Persson M et al. *JAMA Network Open.*

eFigure 5. Regression Standardized Cumulative Incidence of Reintervention, Accounting for the Competing Risk of Death

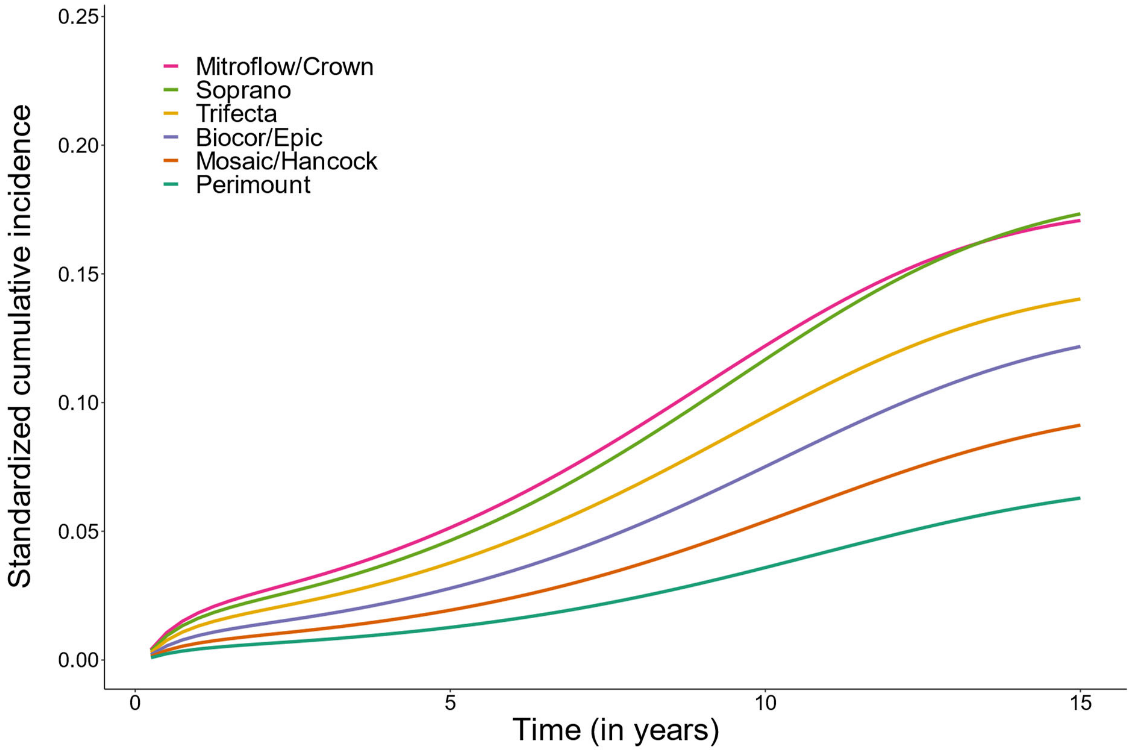

The curves represent the expected outcome if the entire population receives each respective valve group (e.g., if the entire population receives a Perimount valve, 3.6% of the population is expected to have had a reintervention at 10 years).

© 2022 Persson M et al. *JAMA Network Open.*

eFigure 6. Regression Standardized Survival

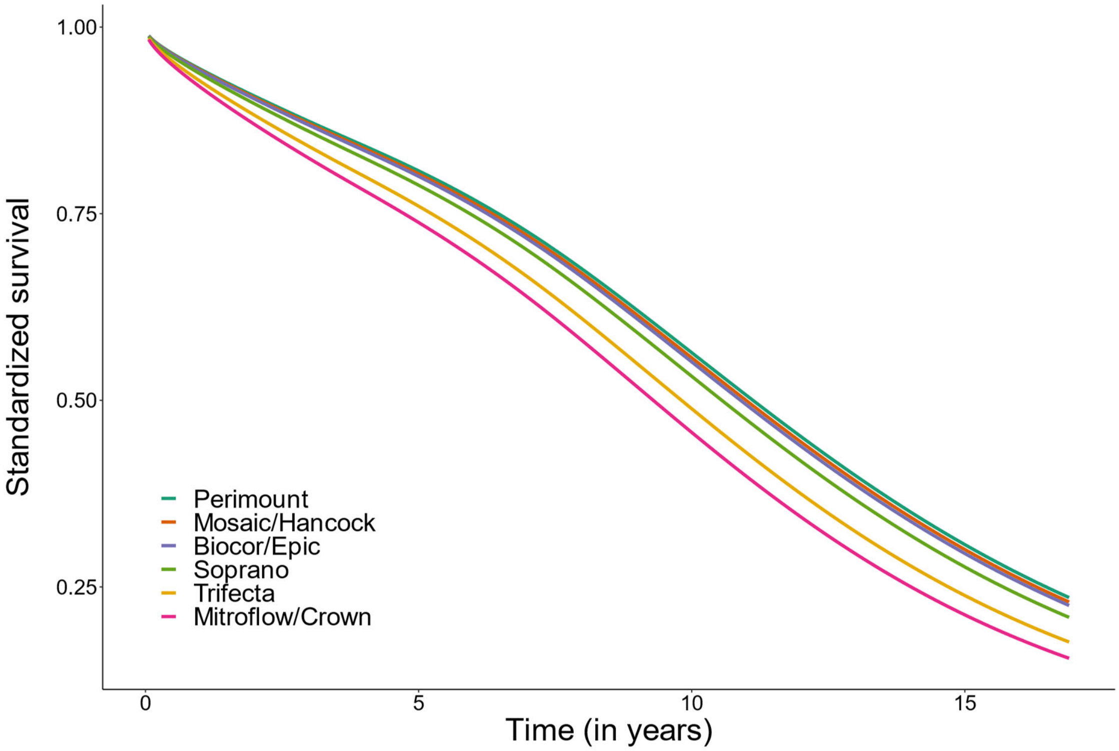

The curves represent the expected survival if the entire population receives each respective valve group (e.g., if the entire population receives a Perimount valve, 56% of the population is expected to be alive at 10 years).

© 2022 Persson M et al. *JAMA Network Open.*

eFigure 7. Regression Standardized Cumulative Incidence of Heart Failure Hospitalization, Accounting for the Competing Risk of Death

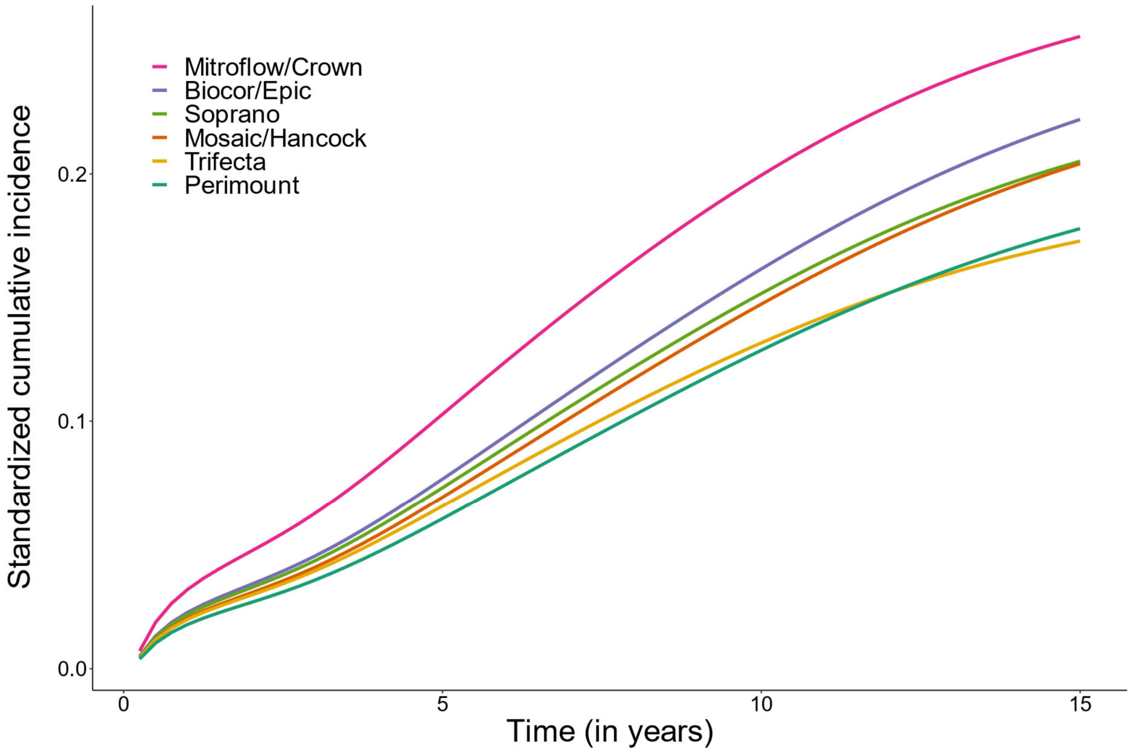

The curves represent the expected outcome if the entire population receives each respective valve group (e.g., if the entire population receives a Perimount valve, 12.9% of the population is expected to have been hospitalized for heart-failure at 10 years).

© 2022 Persson M et al. *JAMA Network Open.*

Analyses on model level

We also performed analyses on model, rather than on model group level. The new groups consisted of Perimount 2900, Perimount Magna (Magna and Magna Ease), Hancock II (Hancock II and Hancock II Ultra), Epic, Biocor, Soprano, Mosaic and Externally mounted valves (Mitroflow, Crown and Trifecta). The results were similar to those obtained from analyses made on model group.

© 2022 Persson M et al. *JAMA Network Open.*

eTable 5. BASELINE CHARACTERISTICS of 16,983 Patients Who Underwent Bioprosthetic Aortic Valve Replacement in Sweden Between 2003 and 2018, Stratified by Model

Variable	Overall	Perimount 2900	Perimount Magna	Hancock II	Epic	Externally mounted	Soprano	Biocor	Mosaic
No.	16983	9279	1990	694	1251	1835	974	419	541
Age, years (mean (SD))	72.6 (8.5)	71.5 (8.9)	70.8 (8.8)	74.5 (7.2)	76.5 (6.3)	74.7 (6.7)	73.5 (8.4)	74.7 (7.2)	75.6 (7.5)
Female sex	6298 (37.1)	3042 (32.8)	1001 (50.3)	219 (31.6)	520 (41.6)	729 (39.7)	359 (36.9)	178 (42.5)	250 (46.2)
Married	10890 (64.1)	5980 (64.4)	1217 (61.2)	433 (62.4)	807 (64.5)	1190 (64.9)	647 (66.4)	279 (66.6)	337 (62.3)
Body mass index, kg/m^2									
<18.5	147 (0.9)	87 (1.0)	15 (0.8)	1 (0.1)	11 (1.1)	18 (1.3)	6 (0.7)	1 (0.3)	8 (1.6)
18.5-24.9	5334 (33.9)	2997 (33.2)	603 (32.7)	231 (33.5)	344 (33.9)	476 (33.3)	318 (38.0)	160 (40.3)	205 (40.4)
25-29.9	6690 (42.5)	3893 (43.2)	706 (38.3)	317 (45.9)	444 (43.7)	605 (42.3)	359 (42.9)	162 (40.8)	204 (40.2)
>30	3562 (22.6)	2037 (22.6)	519 (28.2)	141 (20.4)	216 (21.3)	332 (23.2)	153 (18.3)	74 (18.6)	90 (17.8)
Education									
<10 years	7222 (43.0)	3805 (41.5)	742 (37.6)	290 (42.0)	656 (53.4)	763 (42.1)	448 (46.6)	234 (56.7)	284 (53.7)
10-12 years	6320 (37.7)	3510 (38.3)	803 (40.7)	277 (40.1)	406 (33.0)	685 (37.8)	344 (35.8)	129 (31.2)	166 (31.4)
>12 years	3241 (19.3)	1859 (20.3)	427 (21.7)	124 (17.9)	167 (13.6)	365 (20.1)	170 (17.7)	50 (12.1)	79 (14.9)
Household income									
Q1 (lowest)	4245 (25.0)	2120 (22.8)	433 (21.8)	161 (23.2)	442 (35.4)	447 (24.4)	283 (29.1)	154 (36.8)	205 (37.9)
Q2	4245 (25.0)	2223 (24.0)	449 (22.6)	177 (25.5)	331 (26.5)	462 (25.2)	292 (30.0)	148 (35.3)	163 (30.1)
Q3	4245 (25.0)	2388 (25.7)	526 (26.4)	181 (26.1)	279 (22.3)	482 (26.3)	209 (21.5)	78 (18.6)	102 (18.9)

© 2022 Persson M et al. *JAMA Network Open.*

Q4 (highest)	4245 (25.0)	2547 (27.5)	582 (29.2)	175 (25.2)	198 (15.8)	443 (24.2)	190 (19.5)	39 (9.3)	71 (13.1)
Non-Nordic birth region	1016 (6.0)	533 (5.7)	130 (6.5)	36 (5.2)	75 (6.0)	114 (6.2)	76 (7.8)	24 (5.7)	28 (5.2)
LVEF									
<30%	888 (5.3)	473 (5.1)	83 (4.2)	29 (4.2)	91 (7.3)	97 (5.3)	78 (8.1)	8 (2.1)	29 (5.4)
30%-50%	3725 (22.1)	2047 (22.3)	349 (17.6)	201 (29.0)	279 (22.3)	415 (22.8)	199 (20.6)	100 (25.7)	135 (25.0)
>50%	12225 (72.6)	6671 (72.6)	1551 (78.2)	464 (66.9)	881 (70.4)	1311 (71.9)	690 (71.4)	281 (72.2)	376 (69.6)
Prior myocardial infarction	2858 (16.8)	1415 (15.2)	273 (13.7)	125 (18.0)	296 (23.7)	392 (21.4)	197 (20.2)	59 (14.1)	101 (18.7)
Prior heart failure	3701 (21.8)	1965 (21.2)	378 (19.0)	128 (18.4)	345 (27.6)	432 (23.5)	236 (24.2)	95 (22.7)	122 (22.6)
Prior atrial fibrillation	3202 (18.9)	1737 (18.7)	339 (17.0)	134 (19.3)	258 (20.6)	373 (20.3)	196 (20.1)	92 (22.0)	73 (13.5)
Pacemaker/ICD	467 (2.7)	277 (3.0)	52 (2.6)	20 (2.9)	23 (1.8)	51 (2.8)	23 (2.4)	8 (1.9)	13 (2.4)
Prior PCI	1561 (9.2)	874 (9.4)	184 (9.2)	78 (11.2)	105 (8.4)	188 (10.2)	91 (9.3)	17 (4.1)	24 (4.4)
Hyperlipidemia	3931 (23.1)	2201 (23.7)	498 (25.0)	214 (30.8)	225 (18.0)	433 (23.6)	207 (21.3)	77 (18.4)	76 (14.0)
Hypertension	9786 (57.6)	5284 (56.9)	1287 (64.7)	481 (69.3)	662 (52.9)	1175 (64.0)	525 (53.9)	187 (44.6)	185 (34.2)
Prior endocarditis	816 (4.8)	522 (5.6)	84 (4.2)	20 (2.9)	32 (2.6)	79 (4.3)	38 (3.9)	12 (2.9)	29 (5.4)
Peripheral vascular disease	2243 (13.2)	1193 (12.9)	236 (11.9)	106 (15.3)	205 (16.4)	277 (15.1)	128 (13.1)	24 (5.7)	74 (13.7)
eGFR, mL/min/1.73 m^2									
<30	480 (2.9)	214 (2.3)	63 (3.3)	14 (2.0)	58 (4.7)	88 (4.9)	18 (1.9)	11 (2.6)	14 (2.6)
30-44	1309 (7.8)	706 (7.7)	110 (5.8)	52 (7.5)	126 (10.2)	163 (9.1)	69 (7.2)	31 (7.5)	52 (9.8)
45-59	3120 (18.7)	1726 (18.8)	296 (15.5)	116 (16.7)	265 (21.4)	364 (20.4)	182 (18.9)	72 (17.3)	99 (18.6)

© 2022 Persson M et al. *JAMA Network Open.*

>60	11793 (70.6)	6526 (71.2)	1437 (75.4)	512 (73.8)	787 (63.7)	1167 (65.5)	696 (72.1)	302 (72.6)	366 (68.9)
COPD	1938 (11.4)	953 (10.3)	213 (10.7)	84 (12.1)	171 (13.7)	281 (15.3)	133 (13.7)	37 (8.8)	66 (12.2)
Diabetes mellitus	3791 (22.3)	1988 (21.4)	494 (24.8)	157 (22.6)	274 (21.9)	483 (26.3)	209 (21.5)	94 (22.4)	92 (17.0)
Prior stroke	2105 (12.4)	1072 (11.6)	254 (12.8)	97 (14.0)	199 (15.9)	280 (15.3)	111 (11.4)	39 (9.3)	53 (9.8)
History of cancer	2911 (17.1)	1497 (16.1)	334 (16.8)	116 (16.7)	248 (19.8)	392 (21.4)	196 (20.1)	68 (16.2)	60 (11.1)
Hepatic disease	279 (1.6)	151 (1.6)	34 (1.7)	10 (1.4)	17 (1.4)	32 (1.7)	19 (2.0)	6 (1.4)	10 (1.8)
Alcohol dependence	500 (2.9)	264 (2.8)	70 (3.5)	29 (4.2)	27 (2.2)	61 (3.3)	36 (3.7)	7 (1.7)	6 (1.1)
Period of surgery, years									
2003-2008	4995 (29.4)	2600 (28.0)	242 (12.2)	0 (0.0)	461 (36.9)	265 (14.4)	471 (48.4)	419 (100.0)	537 (99.3)
2009-2013	6085 (35.8)	3108 (33.5)	582 (29.2)	594 (85.6)	736 (58.8)	566 (30.8)	499 (51.2)	0 (0.0)	0 (0.0)
2014-2018	5903 (34.8)	3571 (38.5)	1166 (58.6)	100 (14.4)	54 (4.3)	1004 (54.7)	4 (0.4)	0 (0.0)	4 (0.7)
Size, mm									
19-21	5772 (34.1)	2611 (28.2)	1056 (53.4)	125 (18.1)	420 (33.7)	718 (39.1)	531 (54.7)	96 (23.0)	215 (39.9)
23	6515 (38.5)	3686 (39.8)	601 (30.4)	345 (49.9)	470 (37.7)	727 (39.6)	301 (31.0)	175 (42.0)	210 (39.0)
≥25	4639 (27.4)	2953 (31.9)	321 (16.2)	221 (32.0)	357 (28.6)	389 (21.2)	138 (14.2)	146 (35.0)	114 (21.2)
Concomitant CABG	6453 (38.0)	3326 (35.8)	567 (28.5)	304 (43.8)	586 (46.8)	799 (43.5)	401 (41.2)	203 (48.4)	267 (49.4)
Ascending aortic surgery	1441 (8.5)	878 (9.5)	171 (8.6)	47 (6.8)	92 (7.4)	170 (9.3)	46 (4.7)	9 (2.1)	28 (5.2)
Emergent operation	289 (1.7)	148 (1.6)	36 (1.8)	13 (1.9)	23 (1.8)	44 (2.4)	15 (1.6)	0 (0.0)	10 (1.9)

Numbers are No. (%) unless otherwise noted. SD = standard deviation, Q = quartile, LVEF = left ventricular ejection fraction, ICD = implantable cardioverter defibrillator, PCI = percutaneous coronary intervention, eGFR = estimated glomerular filtration rate, COPD = chronic obstructive pulmonary disease, CABG = coronary artery bypass grafting

© 2022 Persson M et al. *JAMA Network Open.*

eTable 6. Baseline Characteristics of 16,983 Patients Who Underwent Bioprosthetic Aortic Valve Replacement in Sweden Between 2003 and 2018, Stratified by Outcome

Variable	Overall	No Reintervention	Reintervention	Alive	Dead	No heart failure hospitalization	Heart failure hospitalization
No.	16983	16463	520	10252	6731	15033	1950
Age, years (mean (SD))	72.6 (8.5)	72.8 (8.3)	65.1 (11.2)	70.6 (8.8)	75.7 (6.9)	72.3 (8.6)	75.3 (7.0)
Female sex	6298 (37.1)	6143 (37.3)	155 (29.8)	3608 (35.2)	2690 (40.0)	5498 (36.6)	800 (41.0)
LVEF							
<30%	888 (5.3)	861 (5.3)	27 (5.3)	412 (4.0)	476 (7.2)	771 (5.2)	117 (6.1)
30%-50%	3725 (22.1)	3632 (22.2)	93 (18.1)	1943 (19.0)	1782 (26.8)	3178 (21.3)	547 (28.4)
>50%	12225 (72.6)	11832 (72.5)	393 (76.6)	7845 (76.9)	4380 (66.0)	10961 (73.5)	1264 (65.6)
Emergent operation	289 (1.7)	278 (1.7)	11 (2.1)	129 (1.3)	160 (2.4)	265 (1.8)	24 (1.2)
Concomitant CABG	6453 (38.0)	6323 (38.4)	130 (25.0)	3386 (33.0)	3067 (45.6)	5558 (37.0)	895 (45.9)
Ascending aortic surgery	1441 (8.5)	1396 (8.5)	45 (8.7)	1088 (10.6)	353 (5.2)	1358 (9.0)	83 (4.3)
Non-Nordic birth region	1016 (6.0)	967 (5.9)	49 (9.4)	686 (6.7)	330 (4.9)	921 (6.1)	95 (4.9)
Education							
<10 years	7222 (43.0)	7032 (43.2)	190 (36.8)	3769 (37.1)	3453 (52.1)	6268 (42.2)	954 (49.8)
10-12 years	6320 (37.7)	6102 (37.5)	218 (42.2)	4058 (39.9)	2262 (34.1)	5632 (37.9)	688 (35.9)
>12 years	3241 (19.3)	3132 (19.3)	109 (21.1)	2332 (23.0)	909 (13.7)	2969 (20.0)	272 (14.2)

© 2022 Persson M et al. *JAMA Network Open.*

Prior atrial fibrillation	3202 (18.9)	3149 (19.1)	53 (10.2)	1556 (15.2)	1646 (24.5)	2639 (17.6)	563 (28.9)
Alcohol dependence	500 (2.9)	476 (2.9)	24 (4.6)	275 (2.7)	225 (3.3)	441 (2.9)	59 (3.0)
Prior myocardial infarction	2858 (16.8)	2808 (17.1)	50 (9.6)	1395 (13.6)	1463 (21.7)	2412 (16.0)	446 (22.9)
History of cancer	2911 (17.1)	2852 (17.3)	59 (11.3)	1649 (16.1)	1262 (18.7)	2584 (17.2)	327 (16.8)
COPD	1938 (11.4)	1889 (11.5)	49 (9.4)	973 (9.5)	965 (14.3)	1659 (11.0)	279 (14.3)
Diabetes mellitus	3791 (22.3)	3693 (22.4)	98 (18.8)	2027 (19.8)	1764 (26.2)	3241 (21.6)	550 (28.2)
Prior endocarditis	816 (4.8)	763 (4.6)	53 (10.2)	497 (4.8)	319 (4.7)	741 (4.9)	75 (3.8)
Prior heart failure	3701 (21.8)	3616 (22.0)	85 (16.3)	1671 (16.3)	2030 (30.2)	3175 (21.1)	526 (27.0)
Hyperlipidemia	3931 (23.1)	3810 (23.1)	121 (23.3)	2593 (25.3)	1338 (19.9)	3482 (23.2)	449 (23.0)
Hypertension	9786 (57.6)	9536 (57.9)	250 (48.1)	6089 (59.4)	3697 (54.9)	8640 (57.5)	1146 (58.8)
Hepatic disease	279 (1.6)	270 (1.6)	9 (1.7)	130 (1.3)	149 (2.2)	250 (1.7)	29 (1.5)
Peripheral vascular disease	2243 (13.2)	2193 (13.3)	50 (9.6)	1277 (12.5)	966 (14.4)	1987 (13.2)	256 (13.1)
Prior stroke	2105 (12.4)	2053 (12.5)	52 (10.0)	1147 (11.2)	958 (14.2)	1834 (12.2)	271 (13.9)
Pacemaker/ICD	467 (2.7)	463 (2.8)	4 (0.8)	229 (2.2)	238 (3.5)	388 (2.6)	79 (4.1)
Prior PCI	1561 (9.2)	1529 (9.3)	32 (6.2)	970 (9.5)	591 (8.8)	1330 (8.8)	231 (11.8)
Married	10890 (64.1)	10578 (64.3)	312 (60.0)	6714 (65.5)	4176 (62.0)	9652 (64.2)	1238 (63.5)
Model							
Perimount 2900	9279 (54.6)	9063 (55.1)	216 (41.5)	6028 (58.8)	3251 (48.3)	8403 (55.9)	876 (44.9)
Perimount Magna	1990 (11.7)	1956 (11.9)	34 (6.5)	1518 (14.8)	472 (7.0)	1826 (12.1)	164 (8.4)

© 2022 Persson M et al. *JAMA Network Open.*

Hancock II	694 (4.1)	680 (4.1)	14 (2.7)	436 (4.3)	258 (3.8)	625 (4.2)	69 (3.5)
Epic	1251 (7.4)	1211 (7.4)	40 (7.7)	530 (5.2)	721 (10.7)	1024 (6.8)	227 (11.6)
Externally mounted	1835 (10.8)	1749 (10.6)	86 (16.5)	1110 (10.8)	725 (10.8)	1605 (10.7)	230 (11.8)
Soprano	974 (5.7)	890 (5.4)	84 (16.2)	398 (3.9)	576 (8.6)	808 (5.4)	166 (8.5)
Biocor	419 (2.5)	398 (2.4)	21 (4.0)	119 (1.2)	300 (4.5)	327 (2.2)	92 (4.7)
Mosaic	541 (3.2)	516 (3.1)	25 (4.8)	113 (1.1)	428 (6.4)	415 (2.8)	126 (6.5)
Household income							
Q1 (lowest)	4245 (25.0)	4125 (25.1)	120 (23.1)	1836 (17.9)	2409 (35.8)	3574 (23.8)	671 (34.4)
Q2	4245 (25.0)	4127 (25.1)	118 (22.7)	2239 (21.8)	2006 (29.8)	3695 (24.6)	550 (28.2)
Q3	4245 (25.0)	4111 (25.0)	134 (25.8)	2813 (27.4)	1432 (21.3)	3814 (25.4)	431 (22.1)
Q4 (highest)	4245 (25.0)	4097 (24.9)	148 (28.5)	3362 (32.8)	883 (13.1)	3947 (26.3)	298 (15.3)
Body mass index, kg/m^2							
<18.5	147 (0.9)	146 (1.0)	1 (0.2)	67 (0.7)	80 (1.3)	136 (1.0)	11 (0.6)
18.5-24.9	5334 (33.9)	5182 (33.9)	152 (32.5)	3060 (31.9)	2274 (37.0)	4795 (34.4)	539 (30.3)
25-29.9	6690 (42.5)	6485 (42.5)	205 (43.8)	4246 (44.3)	2444 (39.8)	5956 (42.7)	734 (41.3)
>30	3562 (22.6)	3452 (22.6)	110 (23.5)	2218 (23.1)	1344 (21.9)	3068 (22.0)	494 (27.8)
Period of surgery, years							
2003-2008	4995 (29.4)	4780 (29.0)	215 (41.3)	1473 (14.4)	3522 (52.3)	4005 (26.6)	990 (50.8)
2009-2013	6085 (35.8)	5889 (35.8)	196 (37.7)	3628 (35.4)	2457 (36.5)	5352 (35.6)	733 (37.6)

© 2022 Persson M et al. *JAMA Network Open.*

2014-2018	5903 (34.8)	5794 (35.2)	109 (21.0)	5151 (50.2)	752 (11.2)	5676 (37.8)	227 (11.6)
eGFR, mL/min/1.73 m^2							
<30	480 (2.9)	465 (2.9)	15 (2.9)	181 (1.8)	299 (4.5)	424 (2.9)	56 (2.9)
30-44	1309 (7.8)	1287 (7.9)	22 (4.3)	529 (5.2)	780 (11.8)	1086 (7.3)	223 (11.7)
45-59	3120 (18.7)	3072 (19.0)	48 (9.4)	1558 (15.4)	1562 (23.7)	2683 (18.1)	437 (23.0)
>60	11793 (70.6)	11367 (70.2)	426 (83.4)	7841 (77.6)	3952 (59.9)	10608 (71.7)	1185 (62.3)
Size, mm							
19-21	5772 (34.1)	5620 (34.3)	152 (29.3)	3158 (30.9)	2614 (39.0)	5033 (33.6)	739 (38.0)
23	6515 (38.5)	6314 (38.5)	201 (38.8)	3914 (38.3)	2601 (38.8)	5763 (38.5)	752 (38.7)
≥25	4639 (27.4)	4474 (27.3)	165 (31.9)	3152 (30.8)	1487 (22.2)	4187 (27.9)	452 (23.3)

Numbers are No. (%) unless otherwise noted. SD = standard deviation, Q = quartile, LVEF = left ventricular ejection fraction, ICD = implantable cardioverter defibrillator, PCI = percutaneous coronary intervention, eGFR = estimated glomerular filtration rate, COPD = chronic obstructive pulmonary disease, CABG = coronary artery bypass grafting

© 2022 Persson M et al. *JAMA Network Open.*

eTable 7. Crude Cumulative Incidence Of Reintervention, All-Cause Mortality and Heart Failure Hospitalization at 5, 10 and 15 Years Following Bioprosthetic Aortic Valve Replacement in Sweden Between 2003 and 2018, per Model

	5 years	10 years	15 years
Reintervention			
Perimount 2900	1.5 (1.2-1.8)	3.1 (2.6-3.6)	5.1 (4.2-6.0)
Perimount Magna	1.5 (0.9-2.1)	2.5 (1.5-3.6)	NA
Hancock II	1.8 (0.8-2.7)	NA	NA
Epic	1.8 (1.0-2.5)	3.5 (2.4-4.6)	4.2 (2.8-5.6)
Externally mounted	3.3 (2.4-4.3)	9.4 (7.2-11.6)	10.0 (7.6-12.3)
Soprano	3.6 (2.4-4.8)	9.3 (7.3-11.3)	NA
Biocor	2.6 (1.1-4.2)	4.3 (2.4-6.2)	NA
Mosaic	1.1 (0.2-2.0)	3.5 (2.0-5.1)	5.1 (3.0-7.1)
All-cause mortality			
Perimount 2900	18 (18-19)	44 (43-46)	73 (70-75)
Perimount Magna	18 (16-20)	42 (38-46)	72 (56-87)
Hancock II	22 (19-25)	52 (45-59)	NA
Epic	23 (21-25)	57 (54-60)	83 (78-88)
Externally mounted	27 (25-29)	70 (66-74)	91 (87-95)
Soprano	23 (20-26)	53 (50-57)	77 (72-82)
Biocor	27 (23-31)	53 (48-58)	74 (69-79)
Mosaic	22 (18-25)	53 (49-58)	82 (78-86)
Heart failure hospitalization			
Perimount 2900	6.3 (5.8-6.9)	13.7 (12.7-14.6)	20.7 (18.9-22.6)
Perimount Magna	8.3 (6.8-9.7)	15.6 (12.9-18.4)	NA
Hancock II	7.8 (5.8-9.9)	NA	NA
Epic	10.4 (8.7-12.1)	18.9 (16.5-21.3)	25.2 (21.5-28.8)
Externally mounted	9.2 (7.7-10.8)	25.5 (22.0-29.0)	28.5 (24.7-32.3)
Soprano	8.9 (7.1-10.7)	17.3 (14.8-19.8)	NA
Biocor	10.3 (7.4-13.2)	18.6 (14.9-22.3)	NA
Mosaic	8.9 (6.5-11.3)	17.4 (14.2-20.7)	24.5 (20.7-28.3)

Reintervention and rehospitalization were investigated using the Aalen-Johansen estimator and accounted for the competing risk of death. The cumulative incidence of death was estimated using Kaplan-Meier methods. CI = confidence interval, NA = not available.

© 2022 Persson M et al. *JAMA Network Open.*

eTable 8. Incidence Rates of Reintervention, All-Cause Mortality And Heart Failure Hospitalization Following Bioprosthetic Aortic Valve Replacement in Sweden Between 2003 and 2018, Stratified by Model

Model group	Reintervention	All-cause mortality	Heart failure hospitalization
Crude			
Perimount 2900	0.34 (0.29-0.38)	5.6 (5.4-5.8)	1.45 (1.35-1.55)
Perimount Magna	0.36 (0.25-0.50)	4.8 (4.3-5.2)	1.83 (1.56-2.14)
Hancock II	0.32 (0.17-0.54)	6.0 (5.3-6.8)	1.67 (1.30-2.11)
Epic	0.35 (0.25-0.48)	7.8 (7.3-8.4)	2.18 (1.91-2.49)
Externally mounted	0.89 (0.71-1.09)	8.3 (7.7-8.9)	2.57 (2.25-2.92)
Soprano	0.91 (0.73-1.13)	7.6 (7.0-8.2)	1.91 (1.63-2.22)
Biocor	0.41 (0.25-0.62)	8.2 (7.3-9.2)	1.97 (1.59-2.42)
Mosaic	0.35 (0.22-0.51)	8.7 (7.9-9.6)	1.96 (1.63-2.34)
Age- and sex-adjusted			
Perimount 2900	0.32 (0.25-0.39)	6.1 (5.7-6.5)	1.49 (1.27-1.73)
Perimount Magna	0.32 (0.21-0.51)	5.6 (5.0-6.1)	1.97 (1.60-2.43)
Hancock II	0.47 (0.30-0.74)	5.5 (5.2-5.9)	1.53 (1.33-1.77)
Epic	0.58 (0.41-0.82)	6.8 (6.3-7.4)	1.94 (1.66-2.28)
Externally mounted	1.38 (0.78-2.47)	7.8 (6.9-8.8)	2.42 (1.88-3.11)
Soprano	1.08 (0.72-1.61)	7.6 (7.0-8.2)	1.87 (1.56-2.25)
Biocor	0.52 (0.46-0.60)	8.0 (7.8-8.3)	1.87 (1.75-2.00)
Mosaic	0.54 (0.40-0.71)	8.0 (7.5-8.6)	1.90 (1.65-2.18)

Age- and sex-adjusted incidence rates were obtained from a Poisson model. CI = confidence interval.

© 2022 Persson M et al. *JAMA Network Open.*

eTable 9. Adjusted Cumulative Incidence for Reintervention, All-Cause Mortality and Heart Failure Hospitalization at 5, 10 and 15 Years Following Bioprosthetic Aortic Valve Replacement in Sweden Between 2003 and 2018, Stratified by Model

	5 years	10 years	15 years
Reintervention			
Perimount 2900	1.3 (1.1-1.5)	3.6 (3.1-4.3)	6.4 (5.2-7.8)
Perimount Magna	1.2 (0.8-1.7)	3.4 (2.4-4.8)	5.9 (4.1-8.4)
Externally mounted	5 (4-6.3)	11.9 (9.7-14.7)	16.8 (13.9-20.2)
Biocor	2.3 (1.4-3.8)	6.3 (3.9-10)	10.3 (6.7-15.7)
Epic	3.1 (2.1-4.5)	8.3 (5.9-11.6)	13.3 (9.7-18)
Hancock II	1.7 (1-2.9)	4.6 (2.7-7.8)	7.9 (4.8-12.8)
Mosaic	2.2 (1.4-3.4)	6.1 (4-9.3)	10.3 (7-14.9)
Soprano	4.6 (3.5-6)	11.6 (9.1-14.7)	17.2 (13.9-21.3)
All-cause mortality			
Perimount 2900	19 (18-20)	44 (42-45)	69 (67-71)
Perimount Magna	20 (18-21)	44 (42-47)	70 (67-73)
Externally mounted	26 (24-28)	54 (52-56)	78 (76-81)
Biocor	21 (18-23)	46 (42-50)	72 (68-76)
Epic	20 (18-21)	44 (42-47)	70 (67-73)
Hancock II	19 (17-21)	44 (40-47)	69 (65-73)
Mosaic	20 (18-22)	45 (42-48)	70 (67-73)
Soprano	21 (20-23)	47 (44-50)	73 (70-75)
Heart failure hospitalization			
Perimount 2900	5.8 (5.4-6.3)	12.4 (11.5-13.4)	17.3 (15.9-18.7)
Perimount Magna	7.4 (6.3-8.7)	15.4 (13.2-17.9)	20.9 (17.9-24.4)
Externally mounted	10 (8.8-11.3)	19.4 (17.2-21.9)	24.9 (22-28.1)
Biocor	8.2 (6.4-10.5)	17.2 (13.6-21.5)	23.4 (18.6-29.1)
Epic	7.5 (6.4-8.8)	16 (13.8-18.5)	22 (19-25.5)
Hancock II	6.1 (4.8-7.8)	13 (10.2-16.4)	18 (14.2-22.6)
Mosaic	7.3 (6-8.8)	15.5 (13-18.6)	21.7 (18.2-25.8)
Soprano	7.4 (6.2-8.7)	15.3 (13-17.9)	20.7 (17.6-24.2)

Model groups were adjusted by regression standardization. A detailed description and documentation regarding included covariates are available in the Supplemental material. CI = confidence interval.

© 2022 Persson M et al. *JAMA Network Open.*

eFigure 8. Regression Standardized Cumulative Incidence of Reintervention, Accounting for the Competing Risk of Death

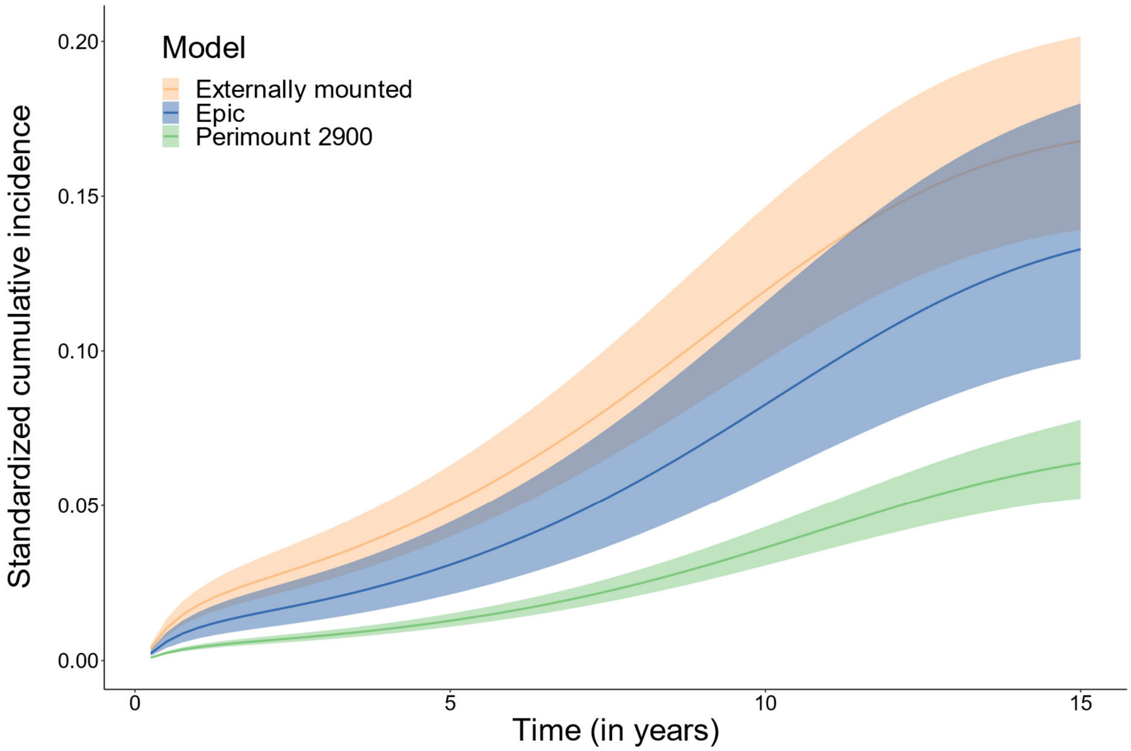

The curves represent the expected outcome if the entire population receives each respective valve group (e.g., if the entire population receives a Perimount valve, 3.6% of the population is expected to have had a reintervention at 10 years).

© 2022 Persson M et al. *JAMA Network Open.*

eFigure 9. Regression Standardized Cumulative Incidence of Reintervention, Accounting for the Competing Risk of Death, All Models

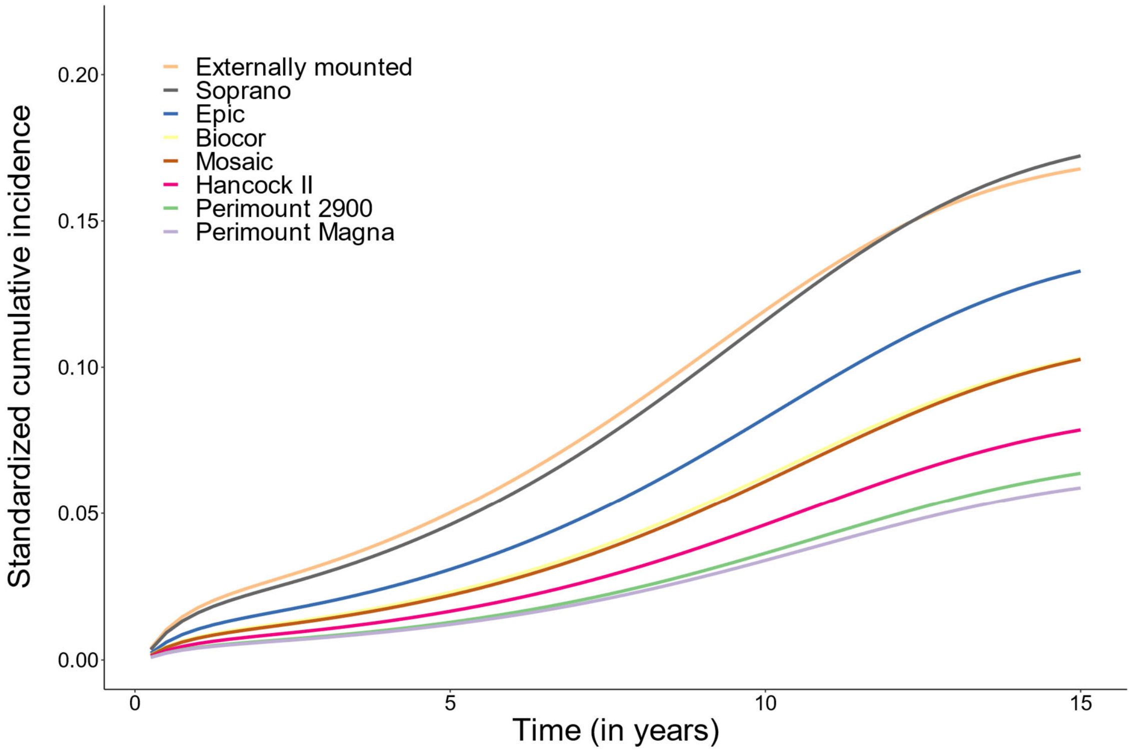

The curves represent the expected outcome if the entire population receives each respective valve group (e.g., if the entire population receives a Perimount valve, 3.6% of the population is expected to have had a reintervention at 10 years).

© 2022 Persson M et al. *JAMA Network Open.*

eFigure 10. Regression Standardized Survival

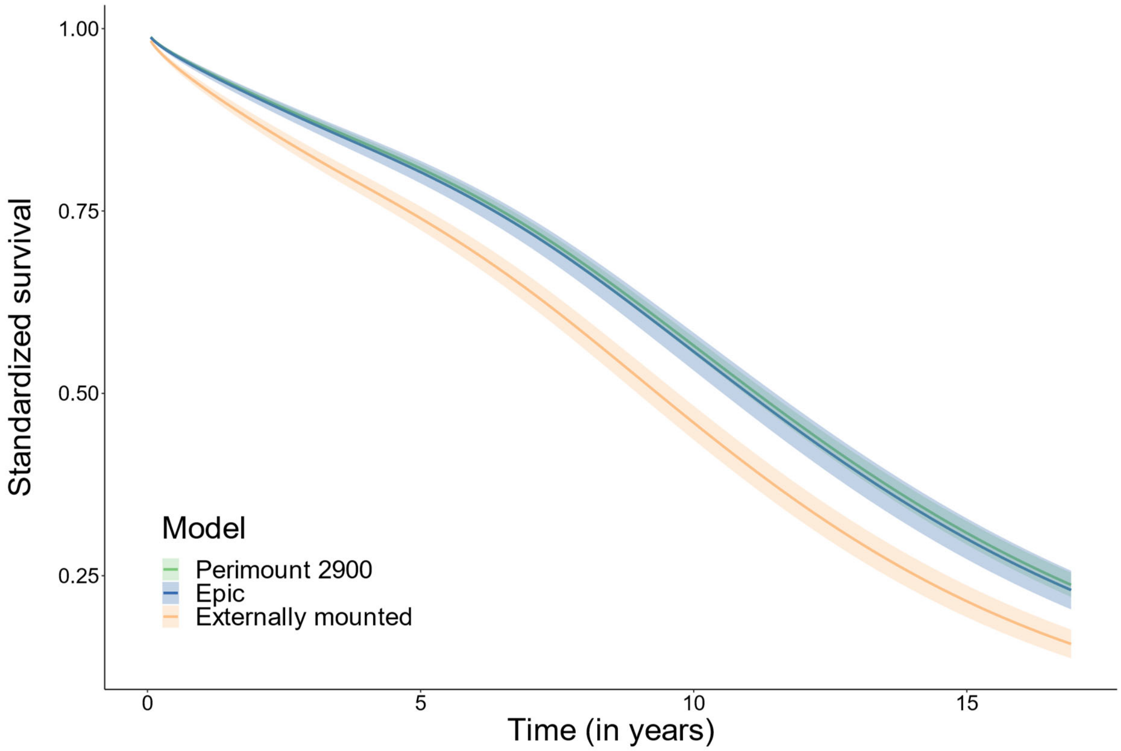

The curves represent the expected survival if the entire population receives each respective valve group (e.g., if the entire population receives a Perimount valve, 56% of the population is expected to be alive at 10 years).

© 2022 Persson M et al. *JAMA Network Open.*

eFigure 11. Regression Standardized Survival, All Models

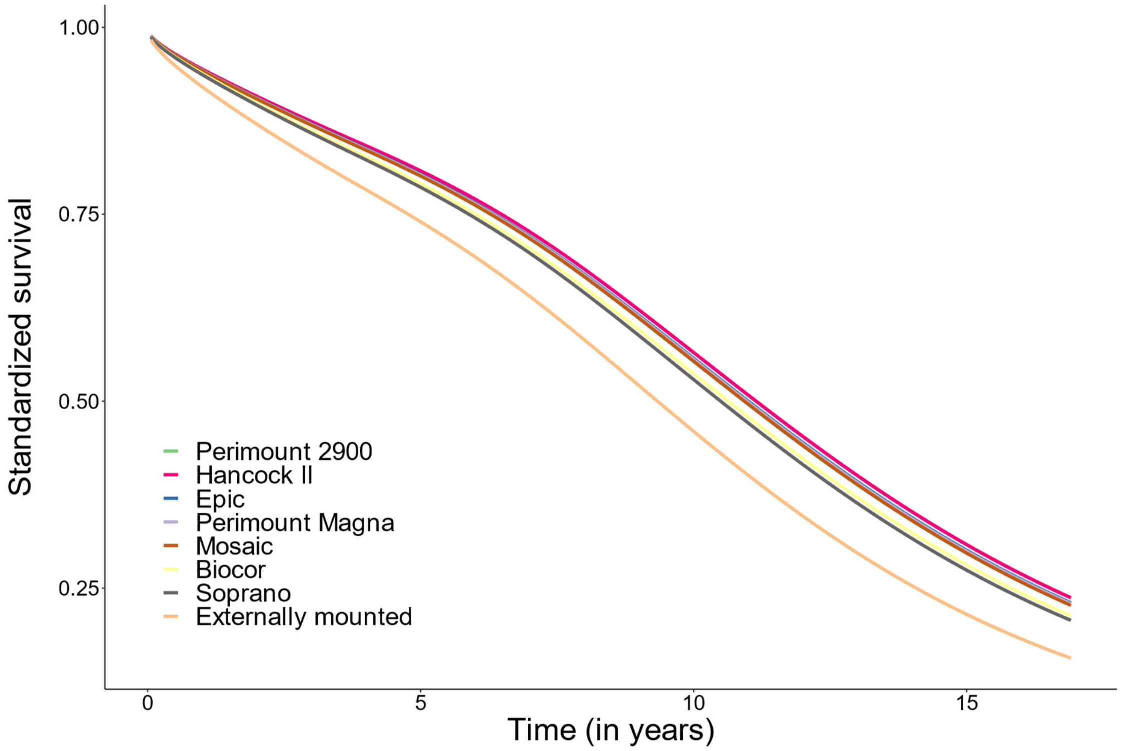

The curves represent the expected survival if the entire population receives each respective valve group (e.g., if the entire population receives a Perimount valve, 56% of the population is expected to be alive at 10 years).

© 2022 Persson M et al. *JAMA Network Open.*

eFigure 12. Regression Standardized Cumulative Incidence of Heart Failure Hospitalization, Accounting for the Competing Risk of Death

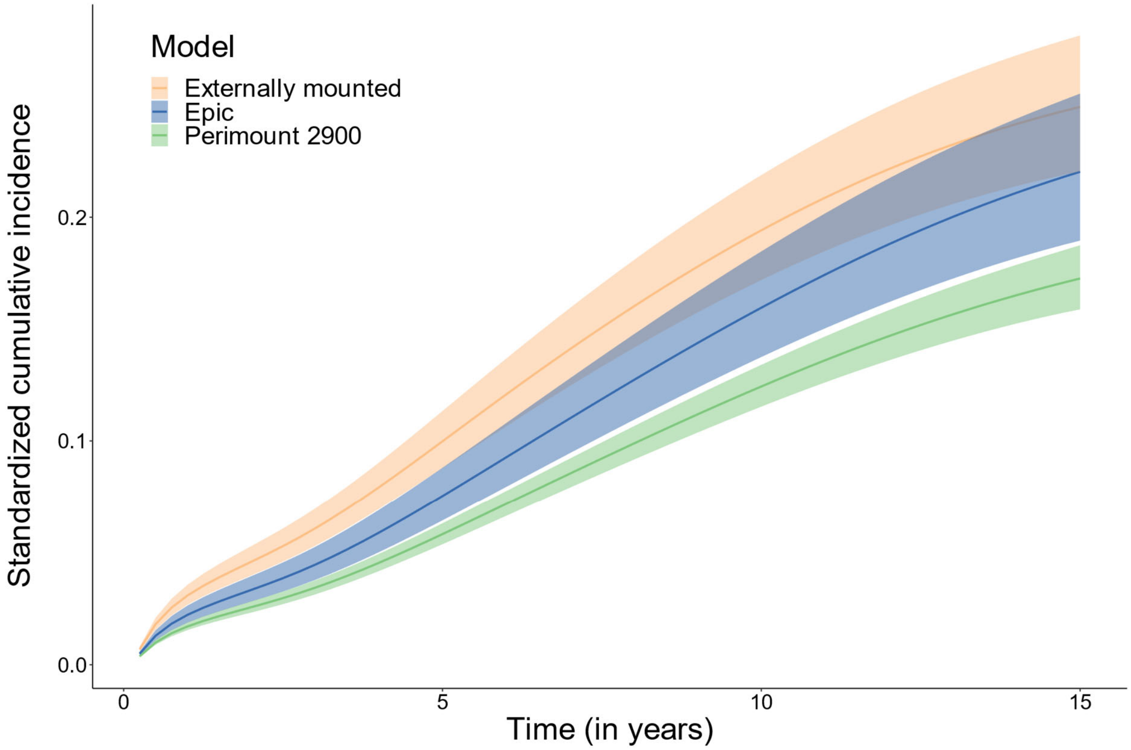

The curves represent the expected outcome if the entire population receives each respective valve group (e.g., if the entire population receives a Perimount valve, 12.4% of the population is expected to be hospitalized for heart-failure at 10 years).

© 2022 Persson M et al. *JAMA Network Open.*

eFigure 13. Regression Standardized Cumulative Incidence of Heart Failure Hospitalization, Accounting for the Competing Risk of Death, All Models

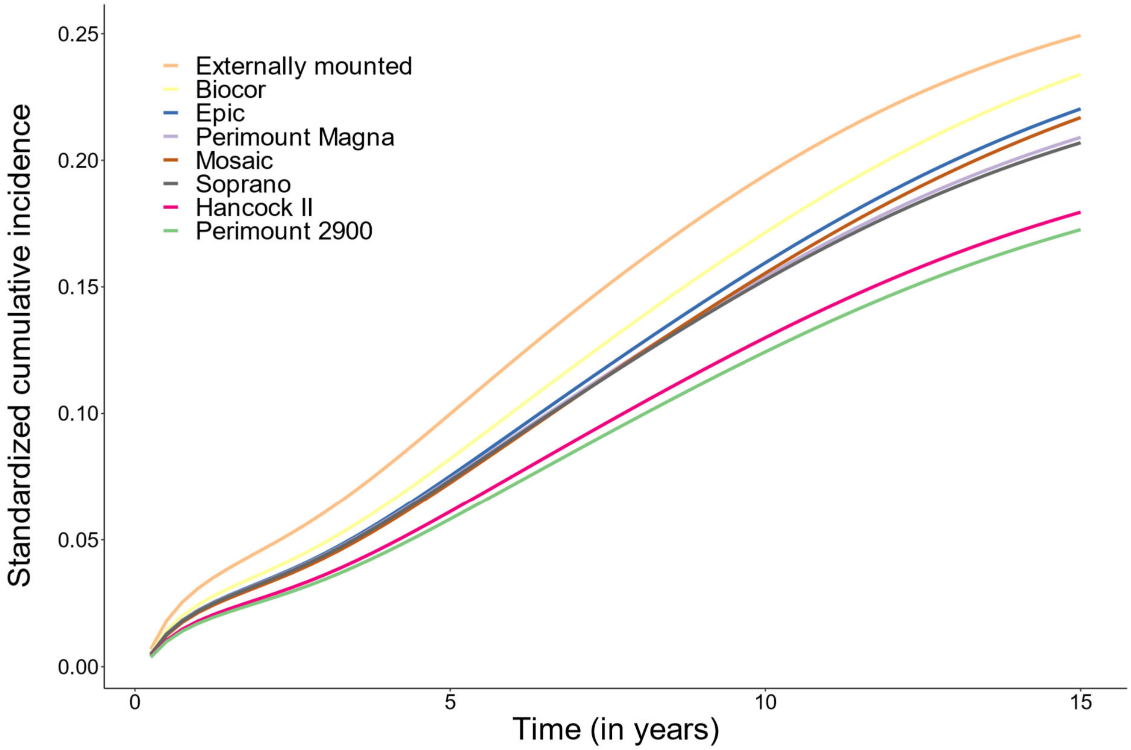

The curves represent the expected outcome if the entire population receives each respective valve group (e.g., if the entire population receives a Perimount valve, 12.4% of the population is expected to be hospitalized for heart-failure at 10 years).

© 2022 Persson M et al. *JAMA Network Open.*
